# Covalent Triazine Framework Nanoparticles via Size‐Controllable Confinement Synthesis for Enhanced Visible‐Light Photoredox Catalysis

**DOI:** 10.1002/anie.202007358

**Published:** 2020-09-21

**Authors:** Wei Huang, Niklas Huber, Shuai Jiang, Katharina Landfester, Kai A. I. Zhang

**Affiliations:** ^1^ Max Planck Institute for Polymer Research Ackermannweg 10 55128 Mainz Germany; ^2^ Department of Materials Science Fudan University 200433 Shanghai P. R. China

**Keywords:** confinement synthesis, covalent triazine frameworks, heterogeneous photocatalysis, nanoparticles, photoredox catalysis

## Abstract

For metal‐free, organic conjugated polymer‐based photocatalysts, synthesis of defined nanostructures is still highly challenging. Here, we report the formation of covalent triazine framework (CTF) nanoparticles via a size‐controllable confined polymerization strategy. The uniform CTF nanoparticles exhibited significantly enhanced activity in the photocatalytic formation of dibenzofurans compared to the irregular bulk material. The optoelectronic properties of the nanometer‐sized CTFs could be easily tuned by copolymerizing small amounts of benzothiadiazole into the conjugated molecular network. This optimization of electronic properties led to a further increase in observed photocatalytic efficiency, resulting in total an 18‐fold enhancement compared to the bulk material. Full recyclability of the heterogeneous photocatalysts as well as catalytic activity in dehalogenation, hydroxylation and benzoimidazole formation reactions demonstrated the utility of the designed materials.

## Graphical abstract

Size‐controllable covalent triazine framework nanoparticles were synthesized through a confined polymerization strategy within silica capsules. They show a manifold increase in photocatalytic activity compared to the bulk system.

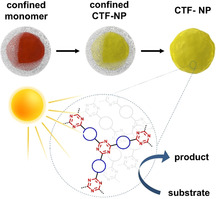

Nanostructured materials have been key to catalytic research in recent years.^1^ By scaling a catalyst down to the nanoscale, the materials surface‐to‐volume ratio increases and active interface is exposed to the reaction media. In most cases, the downscaling has beneficial effects on, for instance, activity, selectivity or turnover of an applied catalyst.^2^ For heterogeneous photocatalytic systems using light as clean and highly sustainable energy source, efforts to synthesize nanometer‐sized morphologies included nanoparticles,^3^ nanowires^4^ as well as hollow nanostructures.^5^ Photocatalysts with improved performance in catalyzing hydrogen evolution from water,^6^ pollutant degradation^7^ or organic reactions^8^ compared to bulk materials have been reported. Clearly, precise morphological control over the material synthesis constitutes a major interest in the production of defined and highly efficient heterogeneous photocatalysts. So far, a vast number of synthetic methods to form nanostructured photocatalysts have been mainly applied to inorganic materials. For pure organic heterogeneous photocatalytic systems, due to their mostly amorphous nature or specific synthetic routes, however, synthesis for defined nanostructures is still highly challenging.

Among the organic photocatalytic systems being used, covalent triazine frameworks (CTFs) have emerged as promising heterogeneous polymer photocatalysts for visible light‐promoted chemical transformations.^9^ They have been employed as efficient platform for visible‐light photoredox reactions such as water splitting,9b, 10 carbon dioxide reduction^11^ or organic reactions.^12^ The CTF systems comprise various advantageous properties, such as high activity in photoredox reactions, low cost and non‐toxicity, excellent recoverability as well as stability in repeated applications. Moreover, CTFs have proven to be easily tunable in their electronic, optical and chemical properties.^13^ However, a main challenge within photocatalytic CTF systems remains in establishing mild synthetic procedures yielding morphologically defined structures. Conventional liquid‐phase approaches in molten ZnCl_2_ or trifluoromethanesulfonic acid (TfOH) solution are not suitable to give CTFs with regular morphologies and defined optical properties. Recently, as one of the few examples, our group reported on the use of silica templates to form hollow or mesoporous CTFs.8b, 14 The group of Tan and Jin was able to synthesize hollow CTF nanospheres for photocatalytic hydrogen evolution.6b The study emphasizes the benefits of nanoscale features for catalytic applications as well as the crucial role of morphology control in designing efficient photoactive CTF materials. Even though initial progress could be made, synthetic methods for self‐standing covalent triazine framework nanoparticles with defined size for photocatalytic applications have not yet been documented.

In this work, we present a confinement synthesis for size‐controllable covalent triazine framework nanoparticles, which is inspired by the baking techniques for fine‐shaped cookies. A thiophene‐derived dinitrile monomer can be encapsulated inside a temporarily formed silica shell using an emulsion approach. Via in situ trifluoromethanesulfonic acid (TfOH)‐catalyzed polymerization followed by silica shell removal, uniform CTF nanoparticles can be obtained. Variation of the emulsifying parameters led to feature sizes between 80 and 550 nm. Apart from controlling the morphology, the electronic and optical properties of the CTF photocatalysts can be further optimized through copolymerizing electron‐withdrawing benzothiadiazole units into the CTF backbone and thereby boosting photogenerated charge separation. Kinetic studies of the photocatalytic dihydrobenzofuran synthesis proved that both nanoscaling and copolymerization had a significant effect on photocatalytic activity with an enhanced 18‐fold efficiency compared to bulk CTF. The photocatalysts’ robustness was underlined by recycling experiments. The versatility of the CTF nanoparticles were further investigated by conducting dehalogenation of α‐chloroacetophenone, hydroxylation of 4‐biphenylboronic acid as well as benzoimidazole formation from *o*‐phenylenediamine with excellent selectivity and efficiency.

The general synthetic route is outlined in Scheme 1 and precisely described in the supplementary material. In detail, the liquid monomer 2,5‐dicyano‐3‐hexylthiophene (DCHT) was specifically designed to ease the formation of a two‐phase system with tetraorthosilicate (TEOS) in water. In aqueous media, DCHT and TEOS combine in a non‐miscible oil phase and can, upon ultrasonication, form a surfactant‐stabilized miniemulsion. When stirred at room temperature, TEOS in the droplets slowly hydrolyses at the oil‐water interphase and forms the silica shell as confinement around the monomer DCHT. The silica encapsulation of liquid monomers in a miniemulsion process was reported in previous studies.^15^ After freeze‐drying, the resulting particles are exposed to trifluoromethanesulfonic acid (TfOH) vapor, leading to formation of the corresponding CTF inside the silica capsule. To obtain pure CTF nanoparticles, the silica shell is removed by etching with a NH_4_HF_2_ solution.

**Scheme 1:**
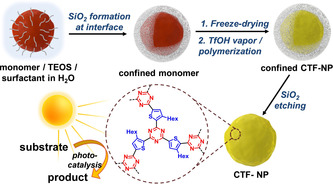
Synthetic route for covalent triazine framework nanoparticles in confinement. In a combined sol–gel emulsion and TfOH vapor‐assisted polymerization approach, CTF nanoparticles are formed. The resulting material can be used for visible light‐promoted organic redox photocatalysis.

To investigate the formation of the nanoparticles, transmission electron microscopy (TEM) as well as scanning electron microscopy (SEM) studies were conducted. As shown in Figure 1 a, TEM imaging indicated the successful confinement of DCHT in the formed silica capsules. A control experiment, in which the premier synthesis step was conducted with *n*‐hexane instead of DCHT yielded collapsed silica capsules after the evaporation of hexane during the freeze‐drying step (Supporting Information, Figure S3). The core–shell structure was retained after the TfOH‐assisted polymerization step as shown in Figure 1 b. Energy‐dispersive X‐ray spectroscopy (EDX) mapping on the encapsulated CTF‐NPs showed high prevalence of silicon and oxygen in the shell area, whereas carbon and sulfur could be detected exclusively in the core of the nanoparticles (Figure 1 c). After the silica shell removal, CTF NPs with different diameters ranging from circa 80 to 550 nm (CTF_80_, CTF_180_, CTF_550_) on average were obtained (Figure 1 d–f; Supporting Information, Figures S22, S23), depending on the volume ratio between dispersed phase and water phase in the miniemulsion synthesis. Light scattering on the colloidal particles in a diluted THF suspension (insert 1d), known as the Tyndall effect, further underlined the successful synthesis of the uniform covalent triazine framework nanoparticles.

**Figure 1:**
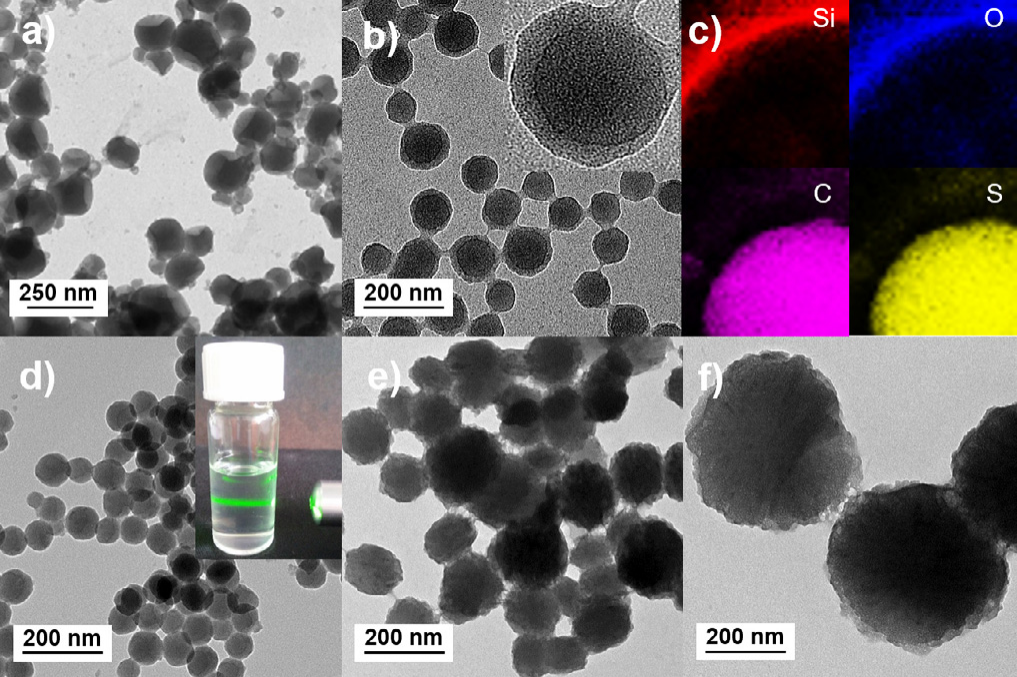
TEM image of a) monomer and b) CTF‐NPs confined in silica capsules. c) Elemental mapping shows enriched contents of silicon and oxygen in the shell and carbon and sulphur in the core. d)–f) Different CTF‐NP sizes of 80, 180 and 550 nm on average could be obtained. The inset in (d) shows the Tyndall effect of CTF_80_ in THF (0.01 mg mL^−1^).

Further characterization was conducted using CTF_80_ (denoted as CTF NPs) if not stated otherwise. The Fourier transformed infrared (FTIR) spectra of the obtained nanoparticles exhibited three characteristic signals of the triazine group at 1482, 1385, and 824 cm^−1^. A comparably low‐intensity signal at 2225 cm^−1^ hints at the presence of terminal nitriles but also indicates the effective formation of triazine networks with high degree of polymerization (Supporting Information, Figure S4). Similar conclusions could be drawn from the corresponding solid‐state ^13^C cross‐polarization magic‐angle‐spinning (CP‐MAS) NMR spectrum. A pronounced signal at ca. 168 ppm verified the presence of sp^2^ triazine carbon atoms (−C=N), whereas the terminal nitrile signal around 112 ppm vanishes into the baseline signal. All signals could be matched with the assumed structure, confirming the CTF synthesis from DCHT (Supporting Information, Figure S5). Powder X‐ray diffraction (PXRD) patterns) showed a broad peak at 13.0° for all materials, which becomes more prominent with increasing BT content (Supporting Information, Figure S6). The general appearance of all CTFs was indeed rather amorphous. Thermogravimetric analysis (TGA) showed that the CTF NPs stay intact up to 400 °C, which is comparable to CTFs from previous studies and proves good thermal stability (Supporting Information, Figure S7).^14^

To further optimize the optoelectronic properties of the as‐synthesized CTF NPs, copolymerizations of DCHT with 1, 2, and 4 mol % of dicyanobenzothiadiazole (DCBT) were carried out. The aim was to incorporate the benzothiadiazole as additional electron acceptor into the CTF backbone structure and further improve the photogenerated charge separation. The resulting CTF NPs were denoted as CTF‐xBT NPs with *x*=1, 2, or 4 depending on the benzothiadiazole (BT) unit content (molar percentage) in the CTF networks. The experimental approach for the nanoparticle synthesis remained unchanged; for experimental details see the supporting information. The morphological nanoparticle formation was confirmed by TEM imaging (Supporting Information, Figure S8). Higher amounts of BT seem to yield less uniform particles after silica removal. FTIR spectra of the CTF‐xBT samples resemble the spectrum of the CTF nanoparticles without BT, hinting at a retained chemical constitution and connectivity of the materials despite the introduction of BT units (Supporting Information, Figure S9). In the ^13^C CP MAS NMR spectra of the CTF‐xBT samples, a minor peak at ca. 155 ppm can be detected, which corresponds to the BT imine carbon in accordance to previous reports (Supporting Information, Figure S10).8b These findings demonstrate the successful introduction of BT units into the conjugated molecular networks.

The optical properties of the CTF nanoparticles were then characterized by UV/Vis diffuse reflection (DR) measurements. As displayed in Figure 2 b, the pristine CTF NPs exhibited visible light absorption which is considered characteristic of semiconductors. Interestingly, the absorption onset at around 550 nm shifts towards longer wavelengths, with increasing the amounts of DCBT. The color of the materials changes with increasing BT content from yellow to brownish orange. This observation could be confirmed by the Kubelka–Munk‐transformed reflectance spectra, which gave band gaps of 2.82 eV for the pristine CTF NPs, 2.79 eV for CTF‐1BT, 2.76 eV for CTF‐2BT and 2.66 eV for CTF‐4BT, respectively (Supporting Information, Figure S11). To assess the location of the lowest unoccupied molecular orbitals (LUMO) of the materials in this study, cyclic voltammetry (CV) measurements were conducted. As depicted in the Supporting Information, Figure S12, a higher BT content shifts the LUMO energy to more positive potentials versus the saturated calomel electrode standard. This trend is consistent with B3LYP/6‐31G(d) density functional theory (DFT) calculations for triazine structures analogous to the CTFs in this study (Supporting Information, Figure S13). By adding the strong electron acceptor 2,1,3‐benzothiadiazole, D‐A charge transfer and therefore electron localization can be further promoted.^16^ The consequent LUMO energy decrease and the reduction of the HOMO/LUMO gap account for the observations made in the optoelectronic characterizations above.

**Figure 2:**
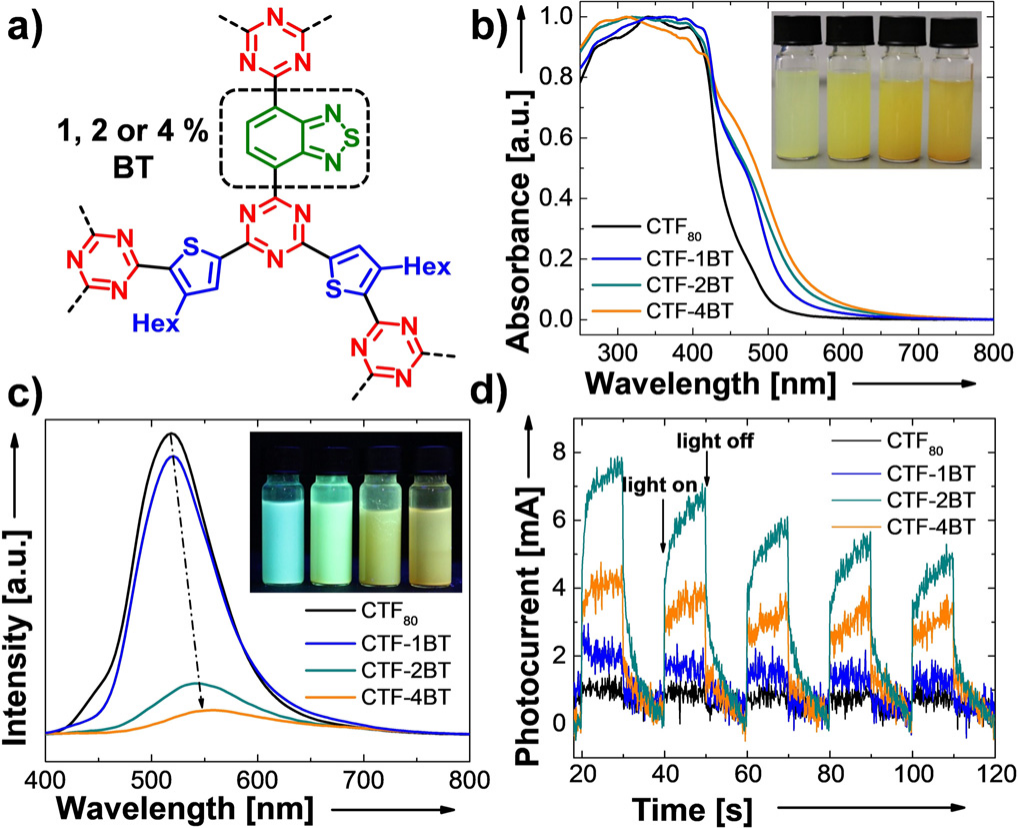
a) Molecular structure, b) diffuse reflectance UV/Vis spectra, c) steady state photoluminescence spectra with *λ*_exc_=380 nm as well as d) photocurrent measurement under visible light irradiation for CTF‐xBT NPs.

Moreover, photoluminescence (PL) spectra with an excitation wavelength of 380 nm were recorded. The PL intensity of the CTF‐xBT NPs significantly decreased with the emission maximum shifting to longer wavelengths compared to the pristine CTF NPs (Figure 2 c). This could be due to a promoted charge carrier separation in CTF‐xBTs leading to other non‐radiative charge recombination pathways being favored. The observed redshift in the catalyst emission characteristics could be derived from the changed UV/Vis absorption behavior. The lowering LUMO levels and band gaps led to the CTF‐xBT NPs emitting from an excited state with lower energy. To further investigate the photogenerated electron separation in CTF‐xBT NPs, photocurrent measurements were conducted (Figure 2 d). A significant increase of photocurrent for CTF‐xBT NPs compared to pristine CTF NPs was observed, with CTF‐2BT showing the highest photocurrent intensity. This indicates an improved light‐induced charge mobility within the copolymerized CTF. Furthermore, electron paramagnetic resonance (EPR) measurements were carried out. As shown in Figure S14, a single Lorentzian line centered at a *g*‐value of 2.0031 was observed for all CTF NPs. This signal originates from unpaired electrons in the π‐conjugated system. Particularly, the EPR intensity of CTF‐2BT showed considerable enhancement compared to the other CTF NPs under visible light irradiation. This might confirm the finding of the photocurrent measurements, where CTF‐2BT could offer more effective photogenerated electrons during the catalytic process. Impedance measurements showed the lowest charge resistance for CTF‐2BT, as displayed in the Supporting Information, Figure S15.

Time‐resolved photoluminescence (TRPL) spectroscopy at an excitation wavelength at 400 nm showed that by adding the strong electron acceptor unit (BT), the fluorescence lifetime decreased proportionally to the increasing BT content (Supporting Information, Figure S16). The lifetimes were 0.89 ns for CTF_80_, 0.57 ns for CTF‐1BT, 0.62 ns for CTF‐2BT and 0.29 ns for CTF‐4BT, respectively. This indicates that among the BT‐containing CTFs, CTF‐2BT might possess the best balance of effective charge separation and delocalization (non‐radiative recombination of the excitons) within the CTF‐xBTs.

We then examined the photocatalytic activity of the CTF NPs in the photocatalytic synthesis of dibenzofuran derivatives. The photocatalytic oxidative [3+2] cycloaddition was first published by Yoon and co‐workers, using ruthenium‐based homogeneous photocatalysts.^17^ The 2,3‐dihydrobenzofuran core is a common motif in bioactive natural products and poses a complex photocatalytic synthesis target.^18^ In a typical experiment, *trans*‐anethol was reacted with mequinol in nitromethane using CTF NPs as photocatalyst under blue light irradiation (Figure 3 a). Ammonium peroxodisulfate was added to act as terminal oxidant. Pristine CTF_80_ NPs exhibited a highly enhanced photocatalytic activity compared to the bulk material (Figure 3 b,c). After 10 h of reaction time, for example, the reaction yield is increased 8‐fold. This effect could be attributed to the decreased particle size and increased number of reactive sites having a positive impact on photocatalytic activity. CTF_180_ and CTF_550_ NPs showed slightly lower activity than CTF_80_. Looking at the copolymerized nanoparticles, CTF‐2BT showed the highest photocatalytic efficiency with a conversion of about 90 % after 10 h, which equals an 18‐ and 2.3‐fold increase compared to the bulk and pristine CTF_80_ NPs, respectively. Considering the similar BET surface area (Supporting Information, Figure S17) and particle size of the CTF‐xBT samples, the superior catalytic efficiency of CTF‐2BT could be attributed to improved photoelectronic properties as indicated previously. The amount of copolymerized electron accepting unit appears to have optimal beneficiary effects on photoelectronic properties at 2 mol % BT, since CTF‐1BT and CTF‐4BT exhibit inferior photocatalytic efficiency in the benchmark reaction. Based on these observations, the nanoscaling (size effect) as well as the introduction of benzothiadiazole into the CTF backbone (electronic effect) synergistically contribute to a substantial improvement in photocatalytic performance.

**Figure 3:**
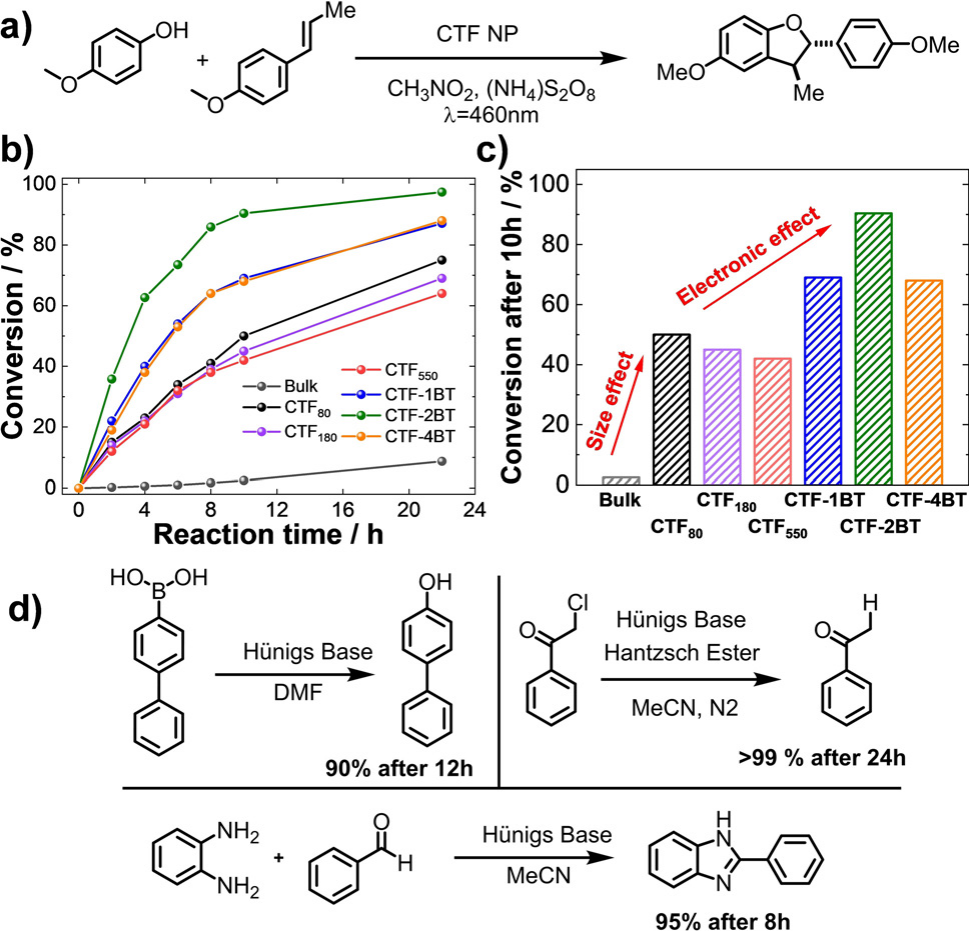
a) Benchmark [3+2] cycloaddition reaction of *trans*‐anethol and mequinol. b) Kinetic study using different CTF nanoparticles as photocatalysts over the course of 24 h and c) at the specific time point of 10 h reaction time. d) Three photoredox reactions catalyzed by CTF‐2BT.

Control experiments conducted in the absence of light, photocatalyst and (NH_4_)_2_S_2_O_8_ only gave trace amounts of product, indicating their indispensable roles in current catalytic system (Supporting Information, Table S1). The catalytic mechanism is proposed in accordance to the literature (Supporting Information, Figure S18) and was supported by scavenger experiments employing CuCl_2_ as electron and KI as hole scavengers.^17^ Upon visible light irradiation, the excited photocatalyst oxidizes the phenol and generates a corresponding radical cation. In the same time, S_2_O_8_^2−^ quenches the generated excited electrons from the LUMO level of the photocatalyst. Through hydrogen abstract transfer (HAT), the radical cation is converted to a resonance‐stabilized phenoxonium cation, which reacts with anethole to give the desired dibenzofuran, after losing one proton. The apparent quantum yield (AQY) in the reaction is wavelength dependent and showed a maximum of 1.89 % (Supporting Information, Figure S19) at 385 nm for CTF‐2BT.

Additionally, recycling experiments demonstrated that CTF‐2BT could be used for five reaction cycles without apparent loss of its catalytic efficiency. No obvious change in FTIR, UV/Vis DR spectra and morphology could be observed (Supporting Information, Figure S20). The heterogeneous photocatalyst therefore reveals excellent stability and recyclability in its photocatalytic application. The versatility of the photocatalytic material was investigated by employing different alkenes and phenols in the cycloaddition reaction (Supporting Information, Figure S21). Apart from depicted synthesis, further organic photoredox reactions could be catalyzed using the nanoparticle catalyst. Furthermore, as shown in the Figure 3 d, dehalogenation of α‐chloroacetophenone, hydroxylation of 4‐biphenylboronic acid as well as benzoimidazole formation from *o*‐phenylenediamine as example of photooxidation, reduction and redox reactions could be catalyzed with excellent yields of above 95 %. This demonstrates general applicability of the CTF nanoparticles in the application as photocatalysts for organic photoredox reactions.

In conclusion, we report the size‐controlled confinement synthesis of covalent triazine framework nanoparticles and their application in organic photoredox catalysis. The CTF nanoparticles were obtained with a uniform size distribution and could be varied in diameter between 80 and 550 nm. Additionally, the optoelectronic properties of the CTF NPs could easily be tuned by copolymerizing electron‐withdrawing benzothiadiazole units. The photocatalytic activity of the CTF NPs was tested in the oxidative [3+2] cycloaddition of *trans*‐anethol and mequinol as benchmark reaction. Both of the decreased particle size and the modified photoelectronic properties through BT‐introduction synergistically contributed to its superior photocatalytic efficiency compared to the bulk CTF material. Moreover, the CTF NPs could be employed as efficient photocatalyst in further dibenzofuran syntheses as well as dehalogenation, hydroxylation and benzoimidazole formation reactions. In summary, the materials depict a compelling example for the morphological, compositional and electronic engineering of advanced polymer‐based photocatalysts. We believe that this study could pave a way for defined nanometer‐sized CTF materials in photocatalytic applications. The innovative and mild assembly route to obtain CTF nanoparticles could be adapted using various monomers and therefore be relevant for a broader application range.

## Conflict of interest

The authors declare no conflict of interest.

## Supporting information

As a service to our authors and readers, this journal provides supporting information supplied by the authors. Such materials are peer reviewed and may be re‐organized for online delivery, but are not copy‐edited or typeset. Technical support issues arising from supporting information (other than missing files) should be addressed to the authors.

Supplementary
