# Supplementary material for: Covalent Triazine Framework Nanoparticles via Size‐Controllable Confinement Synthesis for Enhanced Visible‐Light Photoredox Catalysis
Source: Angew Chem Int Ed Engl. 2020 Sep 21;59(42):18368–73. doi: 10.1002/anie.202007358 (PMC7590189; doi:10.1002/anie.202007358)
Supplement: Supplementary file 1 — Supplementary [file ANIE-59-18368-s001.pdf]

Angewandte Chemie
Eine Zeitschrift der Gesellschaft Deutscher Chemiker

Supporting Information

Covalent Triazine Framework Nanoparticles via Size-Controllable Confinement Synthesis for Enhanced Visible-Light Photoredox Catalysis

Wei Huang[+]*, Niklas Huber*[+]*, Shuai Jiang*[+]*, Katharina Landfester, and Kai A. I. Zhang**

anie_202007358_sm_miscellaneous_information.pdf

Materials

All chemicals and solvents were purchased from commercial sources and used as received unless otherwise noted. Millipore quality (18.2 MΩ cm) water was used throughout.

Characterization

^1H and ^{13}C NMR spectra for all compounds were measured using a Bruker Avance 300 MHz. Solid State ^{13}C CP MAS NMR measurements were carried out using Bruker Avance II solid state NMR spectrometer operating at 300 MHz Larmor frequency equipped with a standard 4mm magic angle spinning (MAS) double resonance probe head. Solid-state diffuse reflectance UV-Vis absorption and fluorescence spectra were recorded on a Perkin Elmer Lambda 100 spectrophotometer and J&M TIDAS spectrofluorometer at ambient temperature, respectively. Gas sorption was measured using a Micromeritics Tristar II Plus with samples degassed for 12 h at 120 ^oC under vacuum prior to analysis. Morphology of nanocapsules was examined with a Gemini 1530 (Carl Zeiss AG, Oberkochem, Germany) scanning electron microscope (SEM) operating at 0.35 kV and a Jeol 1400 (Jeol Ltd, Tokyo, Japan) transmission electron microscope (TEM) operating at an accelerating voltage of 120 kV. SEM and TEM samples of nanocapsules were prepared by casting the diluted dispersion on silicon wafers and carbon layer-coated copper grids, respectively. X-Ray diffraction (XRD) was conducted on a Philips PW 1820 diffractometer with monochromatic Cu Kα radiation. PL lifetime measurements were conducted with a FluoTime200 time-correlated single photon counting setup. Samples were excited with a blue laser at 380 nm which was controlled by PicoQuant PDL 800-D. The signal was detected using a micro-channel plate photomultiplier tube that was connected to PicoHarp 300 time-correlated single photon counting system. PL signals were read at their respective PL emission maxima. Cyclic voltammetry measurements were carried out on a Metrohm Autolab PGSTAT204 potentiostat/galvanostat with a three-electrode-cell system: glassy carbon electrode as the working electrode, $Hg/HgCl_2$ electrode as the reference electrode, platinum wire as the counter electrode, and Bu_4NPF_6 (0.1 M in acetonitrile) as supporting electrolyte with a scan rate of 100 mV s^{-1} in the range of 0 eV to 2 eV. All DFT calculations were carried out with the Gaussian 09 package.[1] The structures were optimized at the B3LYP level of theory[2], with the basis set of 6-31G*.[3] TD-DFT results were obtained from excited state calculations at the same level of theory. Thermogravimetric analysis (TGA) was conducted in a nitrogen atmosphere with temperature increasing from room temperature to 800 °C at a rate of 10 K/min. FT-IR measurements were conducted with a Varian 1000 FT-IR spectrometer. EPR (Electron Paramagnetic Resonance) was measured on a Magnettech Miniscope MS200 spectrometer at room temperature. GC-MS measurements were performed on a Shimadzu GC-2010 plus gas chromatograph and QP2010 ultra mass spectrometer setup (fused silica column Phenomenx, Zebron 5-ms nonpolar, flame ionization detector).

Synthesis of monomers

2,5-Dicyano-3-hexylthiophene: $K_4[Fe(CN)_6]$ * 3 H_2O is dried under vaccum (ca. 0.01mbar) at 60°C for 1h. To a dry 25 mL Schlenk tube was added $K_4[Fe(CN)_6]$ * 3 H_2O (442 mg, 1.20 mmol, 0.40 eq.), CuI (114 mg, 0.60 mmol, 0.20 eq.), 2,5-dibromo-3-hexylthiophene (1.02 g, 3.00 mmol, 1.00 eq.) and 1-methylimmidazole (5 mL). After degassing the solution by Ar bubbling for 20 min, the tube was heated to 140°C for 24h with an oilbath. After cooling to room temperature, the resulting mixture was extracted with dichloromethane (3 x 10 mL). The combined organic phases were washed with brine (30 mL) and dried over anhydrous Na_2SO_4. After filtration and rotary evaporation of solvents the brown oil residue was purified by column chromatography on silica gel (petroleum ethyl acetate / petrol ether 0:1 to 1:10). A colorless oil (256mg, 37%) resulted.

^1H NMR (300 MHz, $CDCl_3$) δ 7.46 (s, 1H), 2.80 (t, 2H), 1.65 (q, 2H), 1.31 (m, 6H) and 0.88 (t, 3H) ppm.

^{13}C NMR (75 MHz, $CDCl_3$) δ 154.59, 137.61, 115.17, 112.36, 112.12, 111.80, 31.45, 30.02, 29.93, 28.77, 22.57 and 14.09 ppm.

Benzo[*c*][1,2,5]thiadiazole-4,7-dicarbonitrile: To a solution of 4,7-dibromobenzothiadiazole (2.0 g, 6.8 mmol) in DMF (10 ml), CuCN (1.8 g, 20.4 mmol) was added. The suspension was degassed with nitrogen for 10 min followed by heating to 145 °C for 20 h. The mixture was allowed to cool to room temperature. Then $FeCl_3 \cdot 6H_2O$ (14 g) in 2M HCl (32 ml) was added into the reaction solution. The resulting suspension was stirred for an hour. The mixture was extracted with DCM (100 mL × 3). The combined organic phase was washed with Milli Q water, dried with anhydrous $MgSO_4$. The filtrate was concentrated under vacuum and purified through a silica column eluting with hexane/ dimethane chloride (1:1) to give the titled product as pale yellow powder (52%).

^1H NMR (300 MHz, $CDCl_3$) δ 8.14 (s, 1H) ppm.

^{13}C NMR (75 MHz, $CDCl_3$) δ 152.56, 134.58, 113.97 and 110.83 ppm.

Preparation of CTF nanoparticles

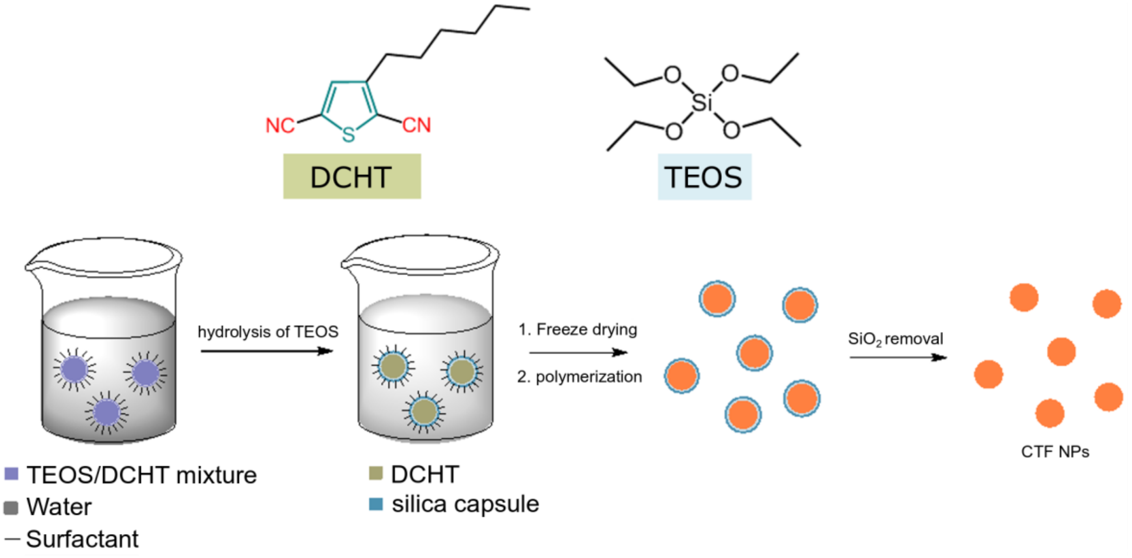

Scheme S1 Synthetic route for covalent triazine framework nanoparticles in confinement.

Step 1: Tetraethoxylsilane (TEOS) (0.52 g, 2.5 mmol) and 2,5-dicyano-3-hexylthiophene (0.4 g, 1.83 mmol) were first mixed to form a homogeneous oil phase. Then an aqueous solution of cetyltrimethylammonium chloride (6.2 mg in 8 mL) was poured into the oil mixture under vigorous stirring. The obtained microemulsion was further sonicated for 3 min using an ultrasonic tip (Branson 450 W, at 70% amplitude). The resulting miniemulsion was stirred at 1000 rpm for another 24 h at room temperature. During this process, the silica capsule was formed by slow hydrolysis of TEOS at the interface of water and the oil phase.
Step 2: The freeze-dried particles were directly polymerized under trifluoromethane sulfonic acid (TfOH) at 100°C in a degassed sealed desiccator for 24 hours. The formed yellow solid was washed with water and diluted aqueous ammonia to remove the TfOH. Further purification was conducted by continuous washing the sample with DCM in a Soxhlet extractor.
Step 3: The resulting yellow powder was stirred with 4M NH_4HF_2, followed by careful washing with water and ethanol. After drying the material at 80°C under vacuum overnight, CTF NPs were obtained as a yellow to orange powder.

Nanoparticles with larger sizes were synthesized by increasing the volume ratio between the dispersed phase and water phase of the emulsion to two and four times.

The copolymerization of benzo[*c*][1,2,5]thiadiazole-4,7-dicarbonitrile with 2,5-dicyano-3-hexylthiophene was conducted according to the same procedure. Correspondingly, 1, 2 or 4 mol% of the BT monomer was added when forming the homogeneous oil phase with TEOS. The synthesis was then continued as described above. The resulting nanoparticle samples are denoted as CTF-1BT, CTF-2BT and CTF-4BT.

In a control experiment, chloroform (260µL, 3.25 mmol) was used instead of DCHT as oil phase. The above procedure was followed. After freeze drying and evaporation of all solvents, TEM pictures (see Figure S3) were taken.

Preparation of bulk CTF

A vial of 2,5-Dicyano-3-hexylthiophene (100 mg) was placed into a conical flask, in which there was another vial with trifluoromethanesulfonic acid (0,3 mL). The conical flask was degassed with nitrogen and sealed followed by heating to 100°C in a sand bath for 24h. After cooling down to room temperature, the product was immersed in water and washed well with ammonia solution (10%) and Milli Q water. The resultant material was further purified with Soxhlet extraction with DCM/MeOH (1:1). After drying under vacuum at 80°C over night, the polymer (48 mg, 48%) was obtained.

Photocatalytic dibenzofurane synthesis

General procedure: To a 20 mL vial with a magnetic stir bar was added nanoparticle photocatalyst (4 mg), alkene (1.30 eq.), phenol (1.0 eq.), $(NH_4)_2S_2O_8$ and nitromethane. Then the vial was capped, degassed for 5 min with nitrogen and placed under the irradiation of a blue LEDs lamp (λ=460 nm, 0.061 W/cm^2). The reaction mixture was stirred at room temperature. The conversion and yield was determined by GC-MS with trimethylsilican as internal standard. Afterwards, the mixture was transferred to a separatory funnel containing DCM and H_2O (v/v, 1/1). The organic

layers were separated and extracted thrice with DCM. The combined organic layers were washed with brine, dried over anhydrous Na_2SO_4 and concentrated by rotary evaporation. The crude product was purified via column chromatography on silica using EtOAc/Hexane as elute to afford the pure compound.

An overview of different dibenzofurane products is shown in Figure S21.

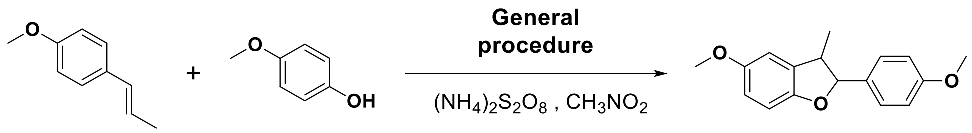

5-methoxy-2-(4-methoxyphenyl)-3-methyl-2,3-dihydrobenzofuran: 4-methoxyphenol (12.4 mg, 0.10 mmol), anethole (0.15 mmol, 22.2 mg), $(NH_4)_2S_2O_8$ (456 mg, 0.20 mmol) and photocatalyst (2 mg) were added into CH_3NO_2 (2 mL). The mixture was irradiated under N_2 for 22 h. The crude product was purified by silica chromatography (EtOAc/Hex=5:95) to afford the product as a colorless oil (26.2 mg, 97%).

^{1}H NMR (300 MHz, $CDCl_3$) δ 7.36 (d, 2H), 6.92 (d, 2H), 6.74 (m, 3H), 5.08 (d, 1H), 3.82 (s, 3H), 3.79 (s, 3H), 3.42 (m, 1h) and 1.38 (d, 3H).

^{13}C NMR (75 MHz, $CDCl_3$) δ 159.79, 154.56, 153.42, 133.26, 132.85, 127.78, 114.15, 113.02, 110.24, 109.48, 92.73, 56.19, 55.45, 45.81 and 17.73 ppm.

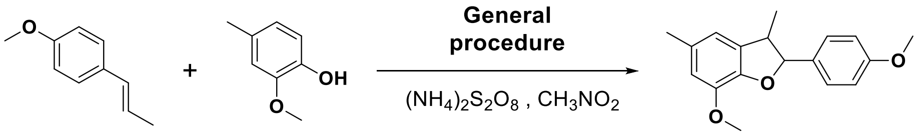

7-methoxy-2-(4-methoxyphenyl)-3,5-dimethyl-2,3-dihydrobenzofuran: 2-methoxy-4-methyl-phenol (13.8 mg, 0.10 mmol), anethole (22.2 mg, 0.15 mmol), $(NH_4)_2S_2O_8$ (456 mg, 0.20 mmol) and photocatalyst (2 mg) were added into CH_3NO_2 (2 mL). The mixture was irradiated under N_2 for 30 h. The crude product was purified by silica chromatography (EtOAc/Hex=10:90) to afford the titled product as a colorless oil (15.1 mg, 53%).

^{1}H NMR (300 MHz, $CDCl_3$) δ 7.40 (d, 2H), 6.93 (d, 2H), 6.64 (d, 2H), 5.15 (d, 1H), 3.91 (s, 3H), 3.84 (s, 3H), 3.47 (m, 1H), 2.33 (s, 3H) and 1.40 (d, 3H) ppm.

^{13}C NMR (75 MHz, $CDCl_3$) δ 159.61, 145.26, 143.86, 133.07, 132.63, 131.01, 127.87, 116.13, 113.87, 112.39, 93.13, 55.99, 55.28, 45.81, 21.31 and 17.79 ppm.

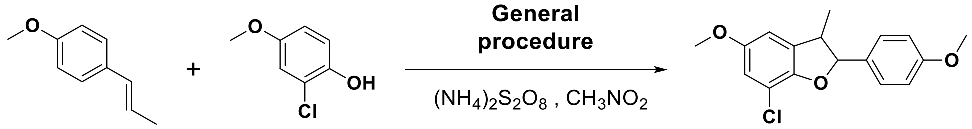

7-chloro-5-methoxy-2-(4-methoxyphenyl)-3-methyl-2,3-dihydrobenzofuran: 2-chloro-4-methoxyphenol (34.5 mg, 0.20 mmol), anethole (44.4 mg, 0.30 mmol), $(NH_4)_2S_2O_8$ (912 mg, 0.40 mmol) and photocatalyst (3 mg) were added into CH_3NO_2 (3 mL). The mixture was

irradiated under N_2 for 30 h. The crude product was purified by silica chromatography (EtOAc/Hex=10:90) to afford the titled product as a colorless oil (16.4 mg, 27%).
^{1}H NMR (300 MHz, $CDCl_3$) δ 7.36 (d, 2H), 6.92 (d, 2H), 6.73 (d, 1H), 6.63 (d, 1H), 5.16 (d, 1H), 3.82 (s, 3H), 3.77 (s, 3H), 3.47 (m, 1H) and 1.38 (d, 3H) ppm.
^{13}C NMR (75 MHz, $CDCl_3$) δ 159.92, 154.81, 149.45, 134.52, 132.06, 127.88, 114.56, 114.16, 113.22, 109.24, 93.16, 56.30, 55.45, 46.52 and 17.75 ppm.

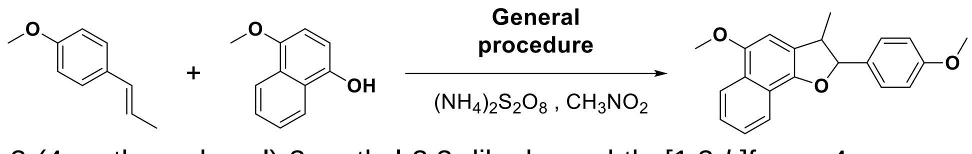

5-methoxy-2-(4-methoxyphenyl)-3-methyl-2,3-dihydronaphtho[1,2-*b*]furan: 4-methoxynaphthalen-1-ol (17.4 mg, 0.10 mmol), anethole (22.2 mg, 0.15 mmol), $(NH_4)_2S_2O_8$ (456 mg, 0.20 mmol) and photocatalyst (2 mg) were added into CH_3NO_2 (2 mL). The mixture was irradiated under N_2 for 12 h. The crude product was purified by silica chromatography (EtOAc/Hex=10:90) to afford the desired product as white solid (31.3 mg, 98%).
^{1}H NMR (300 MHz, $CDCl_3$) δ 8.22 (d, 1H), 7.96 (d, 1H), 7.46 (m, 2H), 7.41 (d, 2H), 6.93 (d, 2H), 6.67 (s, 1H), 5.29 (d, 1H), 3.99 (s, 3H), 3.82 (s, 3H), 3.60 (m, 1H) and 1.48 (d, 3H).
^{13}C NMR (75 MHz, $CDCl_3$) δ 159.72, 150.51, 148.05, 133.65, 127.70, 126.17, 125.66, 125.16, 123.62, 122.63, 121.52, 121.09, 114.17, 100.58, 92.65, 56.25, 55.48, 47.22 and 18.96 ppm.

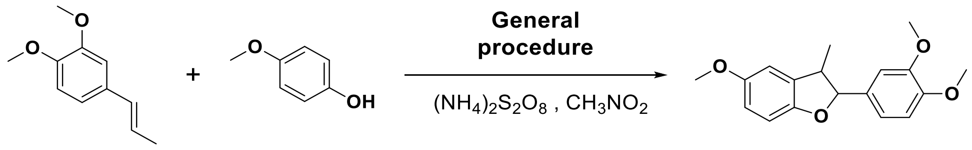

2-(3,4-dimethoxyphenyl)-5-methoxy-3-methyl-2,3-dihydrobenzofuran: 4-methoxyphenol (12.4 mg, 0.10 mmol), trans-methylisoeugenol (35.6 mg, 0.20 mmol), $(NH_4)_2S_2O_8$ (456 mg, 0.20 mmol) and photocatalyst (2 mg) were added into CH_3NO_2 (2 mL). The mixture was irradiated under N_2 for 12 h. The crude product was purified by silica chromatography (EtOAc/Hex=10:90) to afford the desired product as colorless oil (21 mg, 70%).
^{1}H NMR (300 MHz, $CDCl_3$) δ 6.96 (d, 2H), 6.86 (d, 1H), 6.75 (m, 3H), 5.06 (d, 1H), 3.89 (s, 3H), 3.88 (s, 3H), 3.79 (s, 3H), 3.43 (m, 1H) aznd 1.39 (d, 3H) ppm.
^{13}C NMR (75 MHz, $CDCl_3$) δ 157.31, 153.37, 149.41, 149.26, 133.24, 133.16, 119.05, 113.03, 111.13, 110.24, 109.54, 109.35, 93.02, 56.20, 56.09, 56.06, 45.80 and 18.79 ppm.

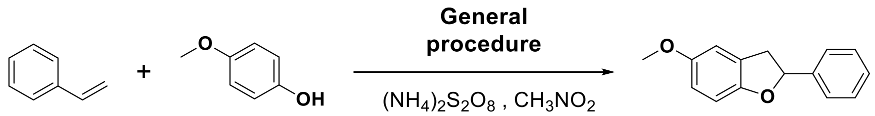

5-methoxy-2-phenyl-2,3-dihydrobenzofuran: 4-methoxyphenol (25.0 mg, 0.20 mmol), styrene (41.6 mg, 0.40 mmol), $(NH_4)_2S_2O_8$ (912 mg, 0.40 mmol) and photocatalyst (3 mg) were added into CH_3NO_2 (3 mL). The mixture was irradiated under N_2 for 12 h. The crude product was

purified by silica chromatography (EtOAc/Hex=5:95) to afford the desired product as colorless oil (23.5 mg, 52%).

^1H NMR (300 MHz, $CDCl_3$) δ 7.38 (m, 5H), 6.79 (m, 2H), 6.72 (m, 1H), 5.75 (t, 1H), 3.78 (s, 3H), 3.61 (m, 1H) and 3.21f (m, 1H) ppm.

^{13}C NMR (75 MHz, $CDCl_3$) δ 154.45, 153.93, 142.16, 128.76, 128.11, 127.63, 125.89, 113.16, 111.35, 109.33, 84.36, 56.17 and 39.01 ppm.

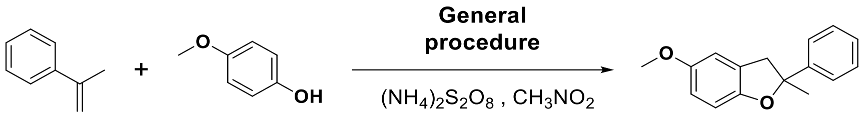

5-methoxy-2-phenyl-2,3-dihydrobenzofuran: 4-methoxyphenol (12.4 mg, 0.10 mmol), α-methylstyrene (23.6 mg, 0.20 mmol), $(NH_4)_2S_2O_8$ (456 mg, 0.20 mmol) and photocatalyst (2 mg) were added into CH_3NO_2 (2 mL). The mixture was irradiated under N_2 for 50 h. The crude product was purified by silica chromatography (EtOAc/Hex=10:90) to afford the desired product as colorless oil (20.1 mg, 84%).

^1H NMR (300 MHz, $CDCl_3$) δ 7.48 (d, 2H), 7.36 (t, 2H), 7.27 (t, 1H), 6.81 (d, 1H), 6.72 (m, 2H), 3.76 (s, 3H), 3.39z (d, 2H) and 1.77 (s, 3H) ppm.

^{13}C NMR (75 MHz, $CDCl_3$) δ 154.30, 153.20, 147.03, 128.48, 127.60, 127.15, 124.67, 113.16, 111.51, 89.32, 56.16, 45.30 and 29.37 ppm.

Photocatalytic recycling experiments

The general procedure was followed for setting up the reactions. After the completion of a reaction cycle after 24h, the conversion was analysed by GCMS and the reaction mixture was centrifuged at 10 000 rpm for 1 min. Then, the supernatant was removed and fresh CH_3NO_2 (2 mL) was added for washing. The centrifugation was repeated and residual CH_3NO_2 was removed. The nanoparticles were then dried in a nitrogen stream for 30 min and weighed. Eventual weight loss was accounted for by adjusting the amounts of *trans*-anethole, mequinol, $(NH_4)_2S_2O_8$ and solvent. After degassing, the next reaction cycle was started by LED irradiation.

Photocatalytic dehalogenation

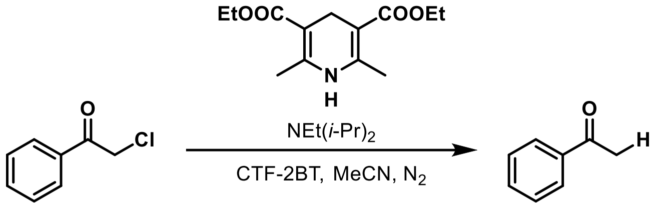

To a 20 mL vial with a magnetic stir bar was added nanoparticle photocatalyst CTF_{2BT} (4 mg), α-chloroacetophenone (77.3 mg, 0.50 mmol, 1.0 eq.), Hünig's base (71.1 mg, 0.55 mmol, 1.1 eq.), Hantzsch ester (253 mg, 1.0 mmol, 2.0 eq.) and acetonitrile (4 mL). Then the vial was capped, degassed by N_2 bubbling and placed under the irradiation of a blue LEDs lamp (0.061 W/cm^2). The reaction mixture was stirred at room temperature for 8h. The GCMS conversion was found to be 99%. Afterwards, the mixture was transferred to a separatory funnel containing DCM and H_2O (v/v, 1/1). The organic layers were separated and extracted thrice with DCM. The combined organic layers were washed with brine, dried over anhydrous $MgSO_4$ and concentrated by rotary evaporation. The crude product was purified via column chromatography (Petrol ether / ethyl acetate, 6:1) on silica to afford benzaldehyde (57 mg, 95%).
^{1}H NMR (300 MHz, $CDCl_3$) δ 10.02 (s, 1H), 7.88 (d, 2H), 7.64 (t, 2H), 7.53 (t, 2H) ppm.
^{13}C NMR (75 MHz, $CDCl_3$) δ 188.45, 136.92, 134.59, 129.87, 129.12 ppm.

Photocatalytic hydroxylation of boronic acids

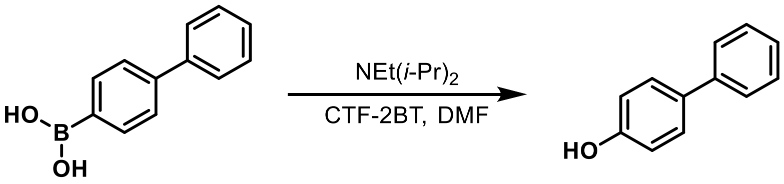

To a 20 mL vial with a magnetic stir bar was added nanoparticle photocatalyst CTF_{2BT} (4 mg), 4-biphenylboronic acid (99.0 mg, 0.50 mmol, 1.0 eq.), Hünig's base (129 mg, 1.0 mmol, 2.0 eq.) and *N,N*-dimethylformamide (4 mL). Then the vial was capped and placed under the irradiation of a blue LEDs lamp (0.061 W/cm^2). The reaction mixture was stirred at room temperature for 12h. The GCMS conversion was found to be 90%. Afterwards, the mixture was transferred to a separatory funnel containing DCM and H_2O (v/v, 1/1). The organic layers were separated and extracted thrice with DCM. The combined organic layers were washed with brine, dried over anhydrous $MgSO_4$ and concentrated by rotary evaporation. The crude product was purified via column chromatography (Petrol ether / ethyl acetate, 3:1) on silica to afford 4-phenylphenol (71 mg, 83%).
^{1}H NMR (300 MHz, $CDCl_3$) δ 7.55 (d, 2H, 7.49 (d, 2H), 7.42 (t, 2H), 7.31 (t, 1H), 6.91 (d, 2H), 4.92 (s, 1H) ppm.
^{13}C NMR (75 MHz, $CDCl_3$) δ 152.67, 140.89, 135.59, 128.87, 128.54, 127.36, 115.78 ppm.

Photocatalytic benzoimidazole formation

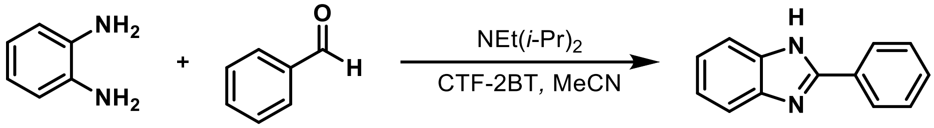

To a 20 mL vial with a magnetic stir bar was added nanoparticle photocatalyst CTF_{2BT} (4 mg), *o*-phenylenediamine (54.1 mg, 0.50 mmol, 1.0 eq.), benzaldehyde (53.1 mg, 0.50 mmol, 1.0 eq.), Hünig's base (129 mg, 1.0 mmol, 2.0 eq.) and acetonitrile (4 mL). Then the vial was capped, degassed by N_2 bubbling and placed under the irradiation of a blue LEDs lamp (0.061 W/cm^2). The reaction mixture was stirred at room temperature for 15h. The GCMS conversion was found to be 95%. Afterwards, the mixture was transferred to a separatory funnel containing DCM and H_2O (v/v, 1/1). The organic layers were separated and extracted thrice with DCM. The combined organic layers were washed with brine, dried over anhydrous $MgSO_4$ and concentrated by rotary evaporation. The crude product was purified via column chromatography (Petrol ether/Ethyl acetate, 1:1, 3% of acetic acid was added to the ethyl acetate used) on silica to afford 2-phenylbenzimidazole (71 mg, 73%) .

^{1}H NMR (300 MHz, $CDCl_3$) δ 8.06 (m, 2H), 7.65 (m, 2H), 7.48 (m, 3H), 7.28 (m, 2H), 4.46 (br s, 1H) ppm.

^{13}C NMR (75 MHz, $CDCl_3$) δ 151.65, 139.09, 130.43, 129.81, 129.26, 126.71, 123.22, 115.36 ppm.

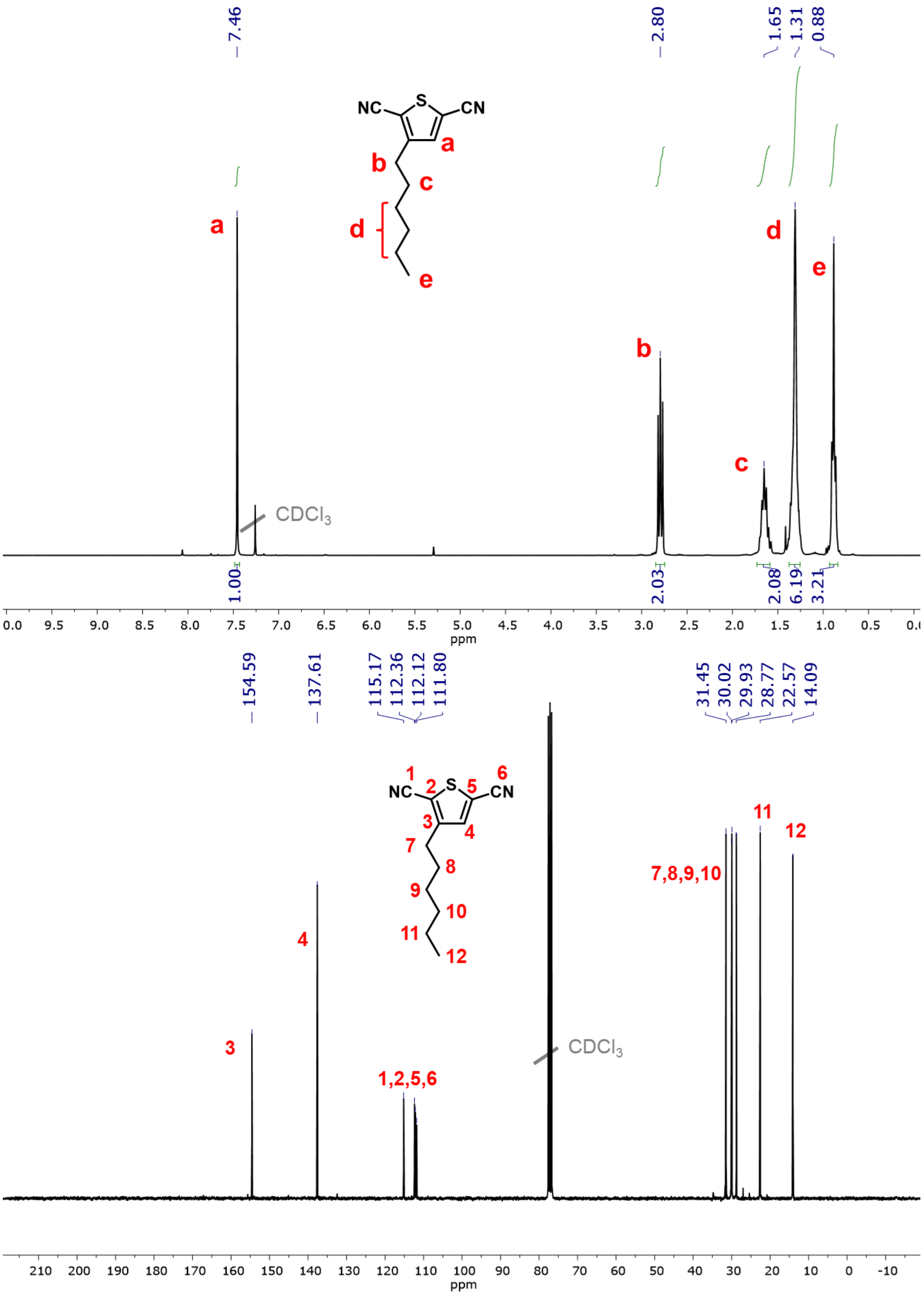

Figure S1. ^{1}H and ^{13}C NMR spectrum of monomer 2,5-dicyano-3-hexylthiophene (300 MHz, $CDCl_3$, at 298 K and 75 MHz, $CDCl_3$, at 298 K).

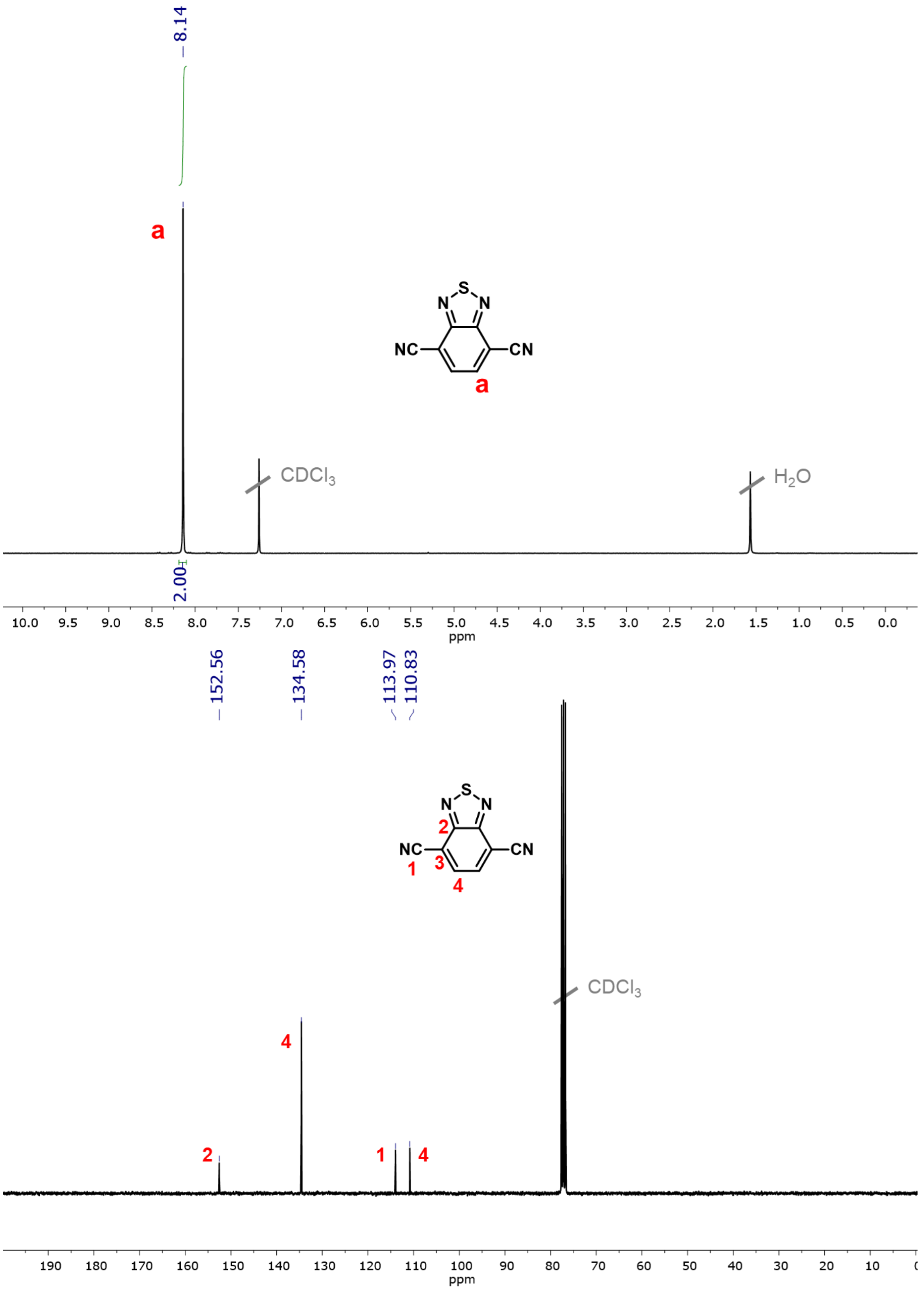

Figure S2. ${}^{1}H$ and ${}^{13}C$ NMR spectrum of monomer benzo[*c*][1,2,5]thiadiazole-4,7-dicarbonitrile (300 MHz, $CDCl_3$, at 298 K and 75 MHz, $CDCl_3$, at 298 K).

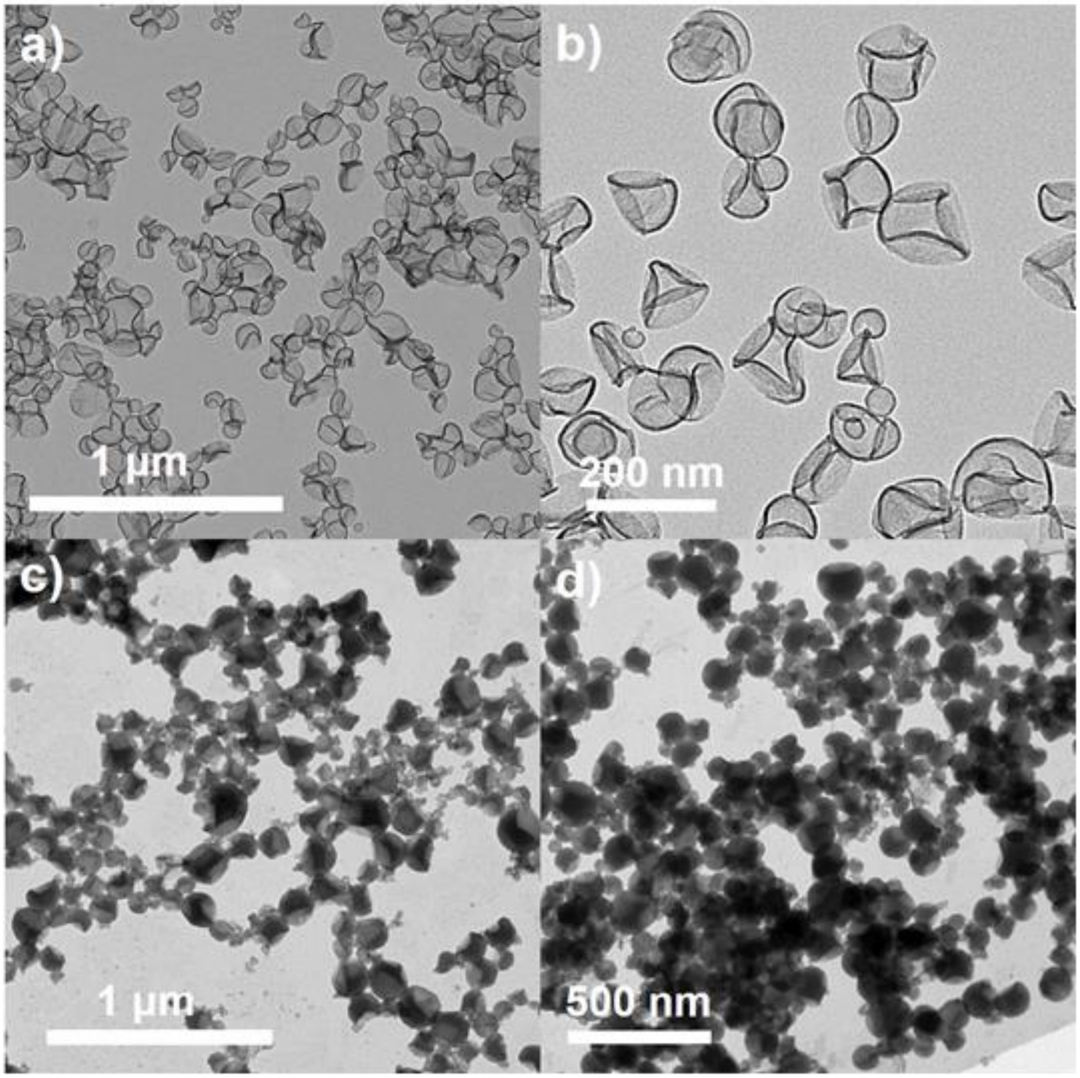

Figure S3. TEM images of silica capsules synthesized without DCHT (a, b) and silica capsules with DCHT (c, d).

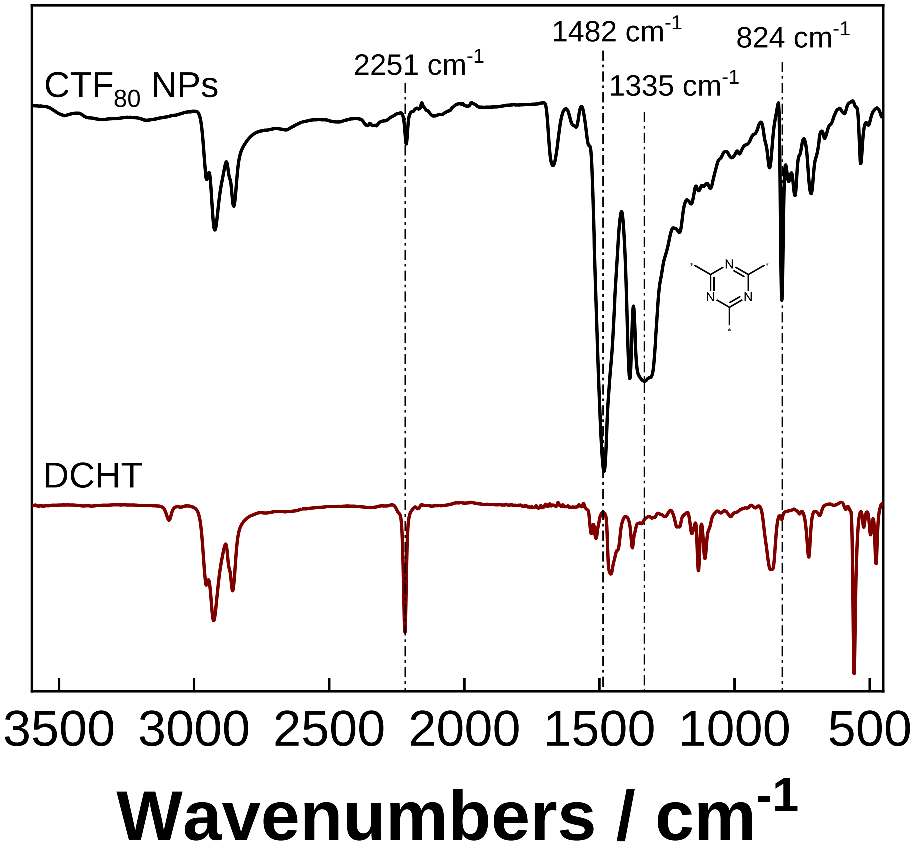

Figure S4. Comparison of Fourier transform infrared (FTIR) spectra of CTF-NPs with the monomer 2,5-dibromo-3-hexylhiophene.

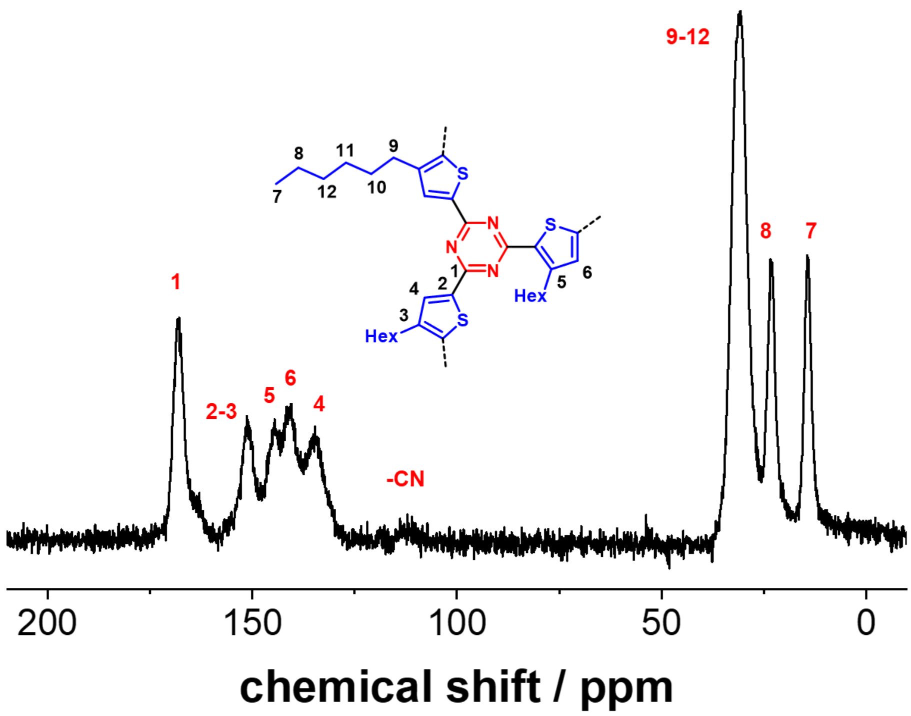

Figure S5. ^{13}C CP-MAS NMR spectrum of CTF_{80} NPs

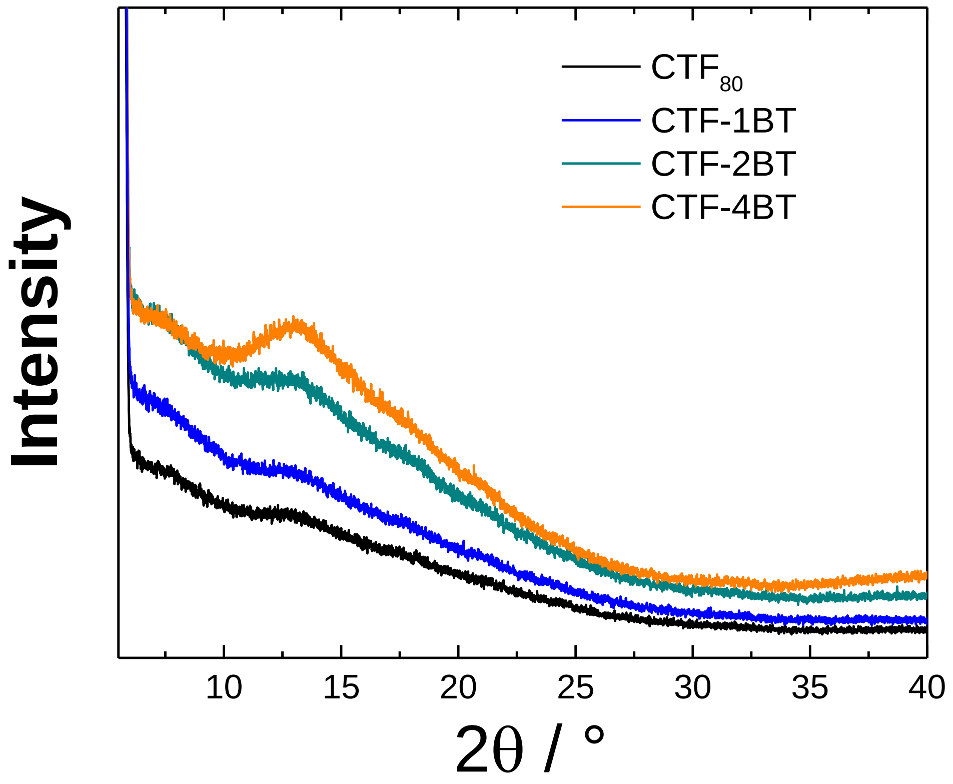

Figure S6. Powder X-ray diffraction (XRD) pattern of CTF_{80}, CTF-1BT, CTF-2BT and CTF-4BT.

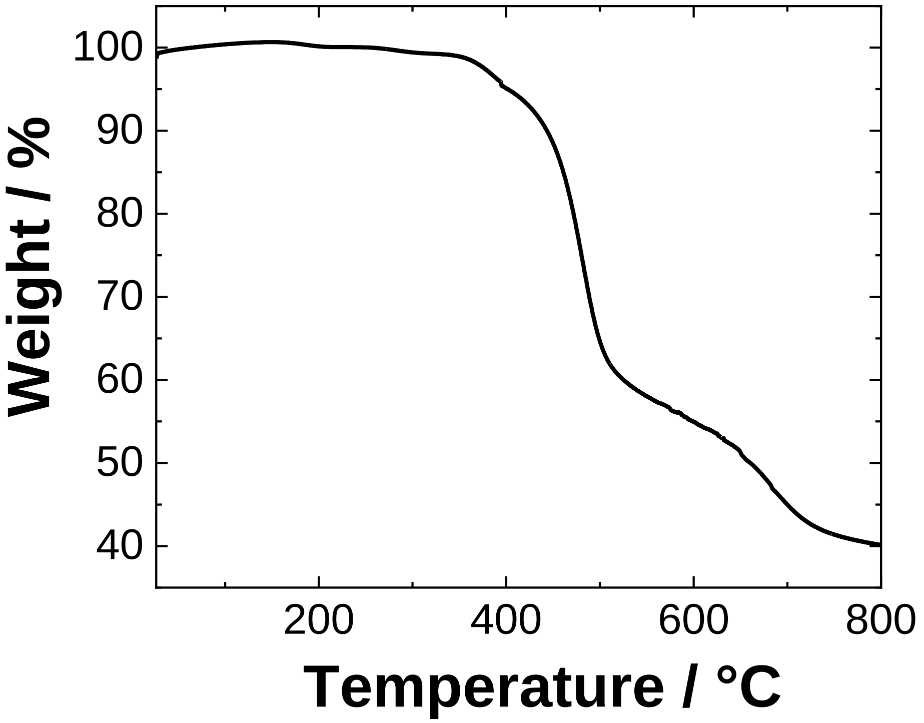

Figure S7. Thermogravimetric analysis (TGA) of CTF_{80} NPs under N_2 with temperature increasing from room temperature to 800°C at a rate of 10 K/min.

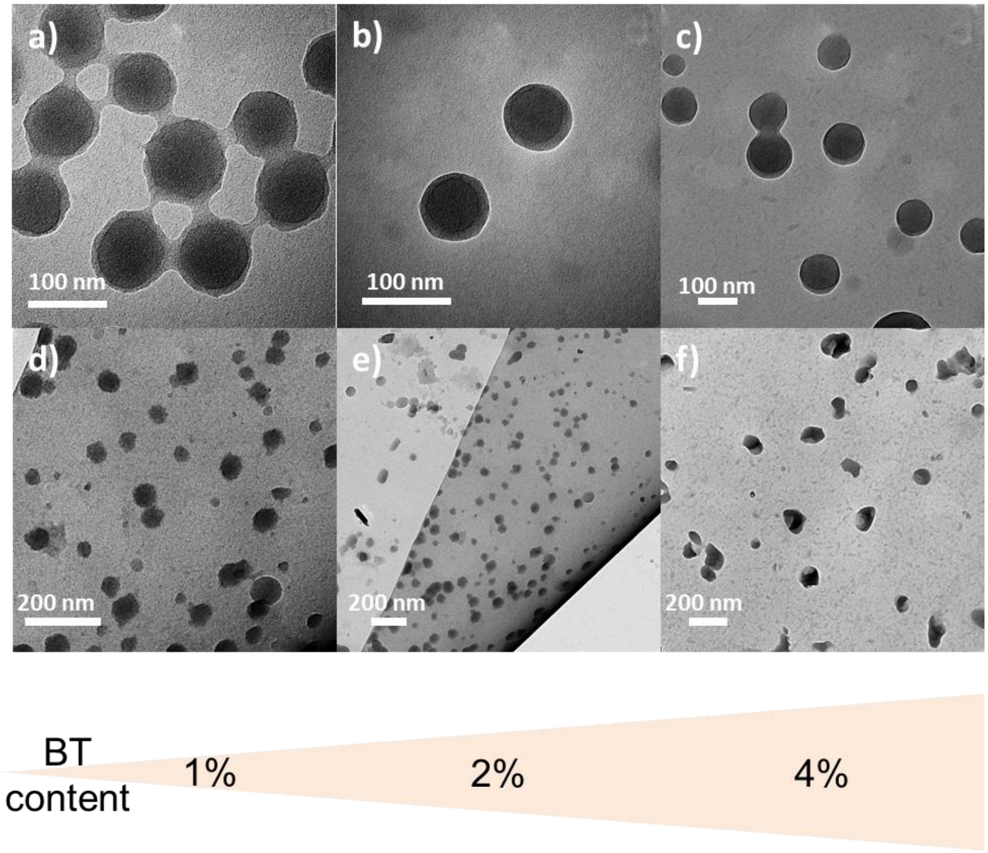

Figure S8. Transmission electron microscopy of CTF-NPs with different BT contents in silica capsules (a-c) and after removing silica (d-f).

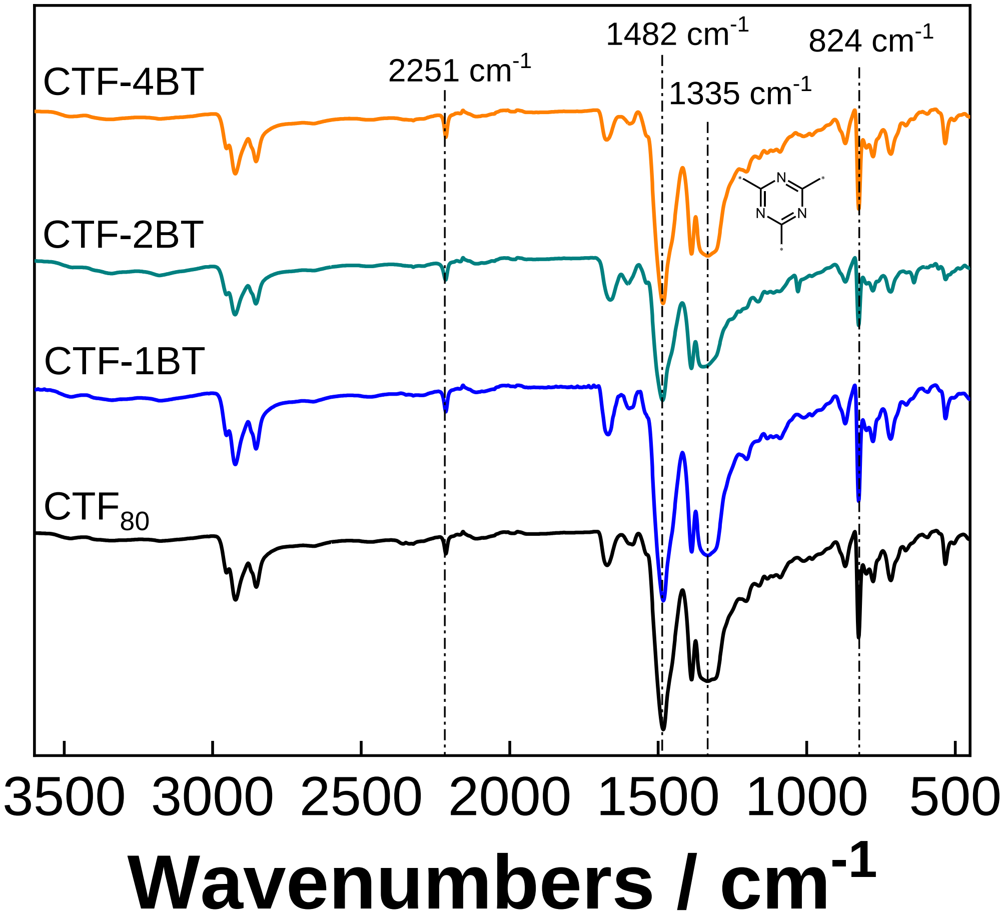

Figure S9. Comparison of Fourier transform infrared (FTIR) spectra of CTF-NPs with different BT content.

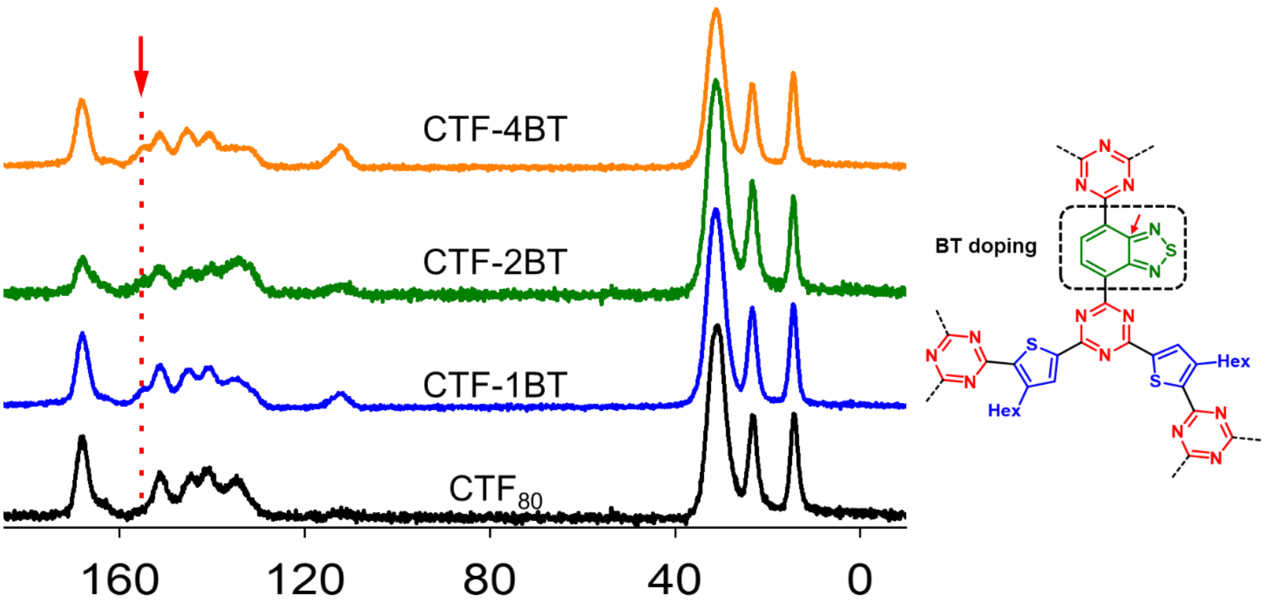

Figure S10. Comparison of ^{13}C CP-MAS NMR spectrum of CTF-NPs with different BT content.

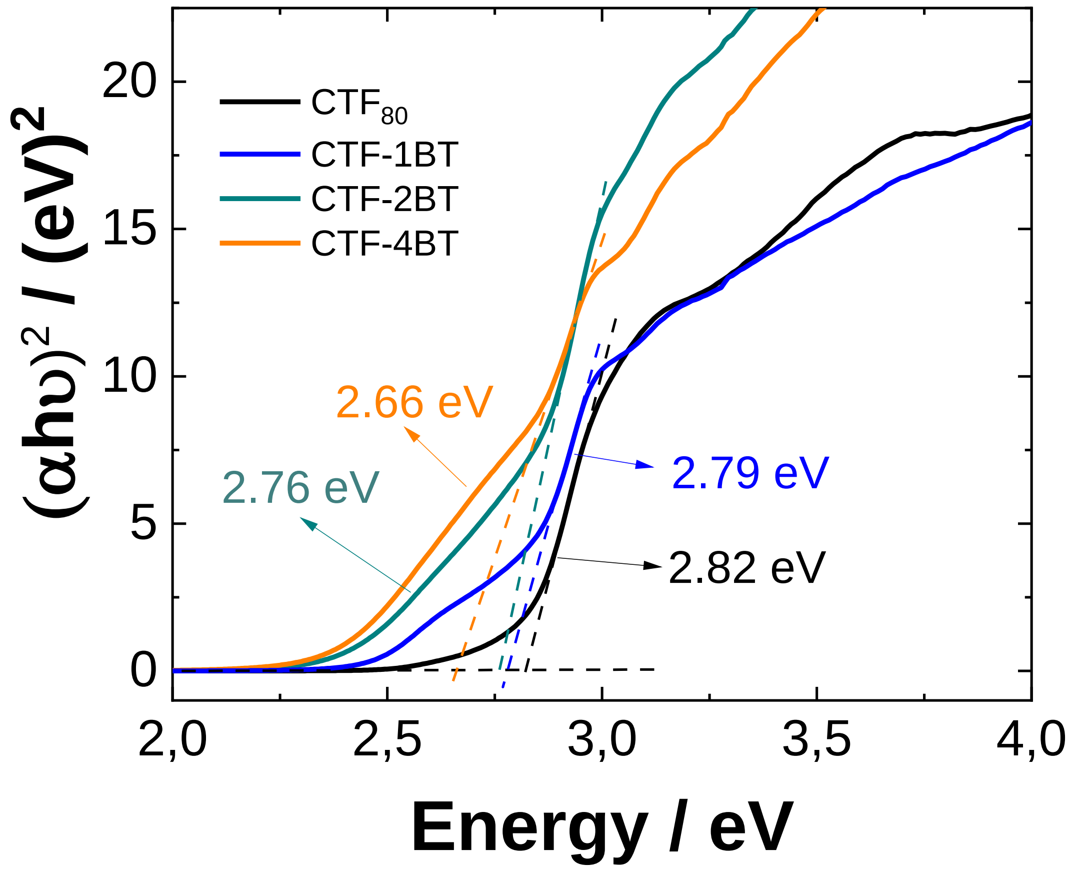

Figure S11. Kubelka-Munk transformed UV/Vis reflectance spectra of CTF nanoparticles with different BT contents.

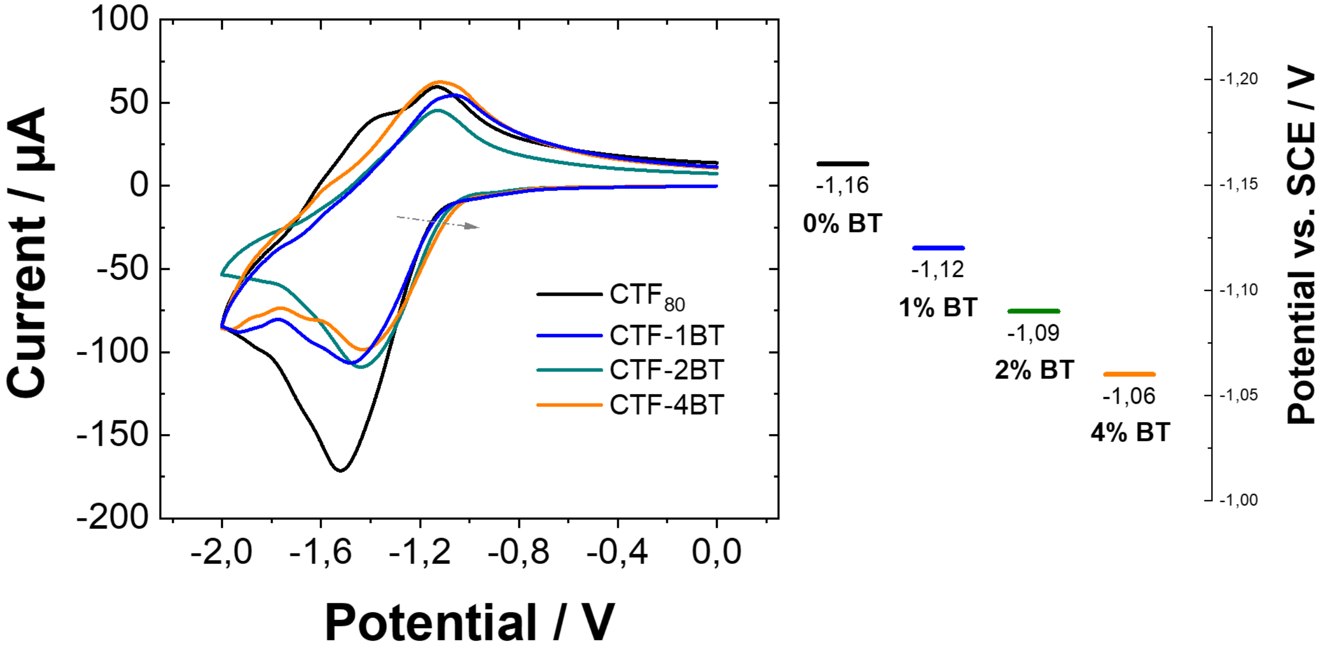

Figure S12. Cyclic voltammetry of CTF-NPs with different BT contents with a scan rate of 100 mV/s in CH_3CN and CV HOMO potentials vs. SCE as deducted from the onset potential.

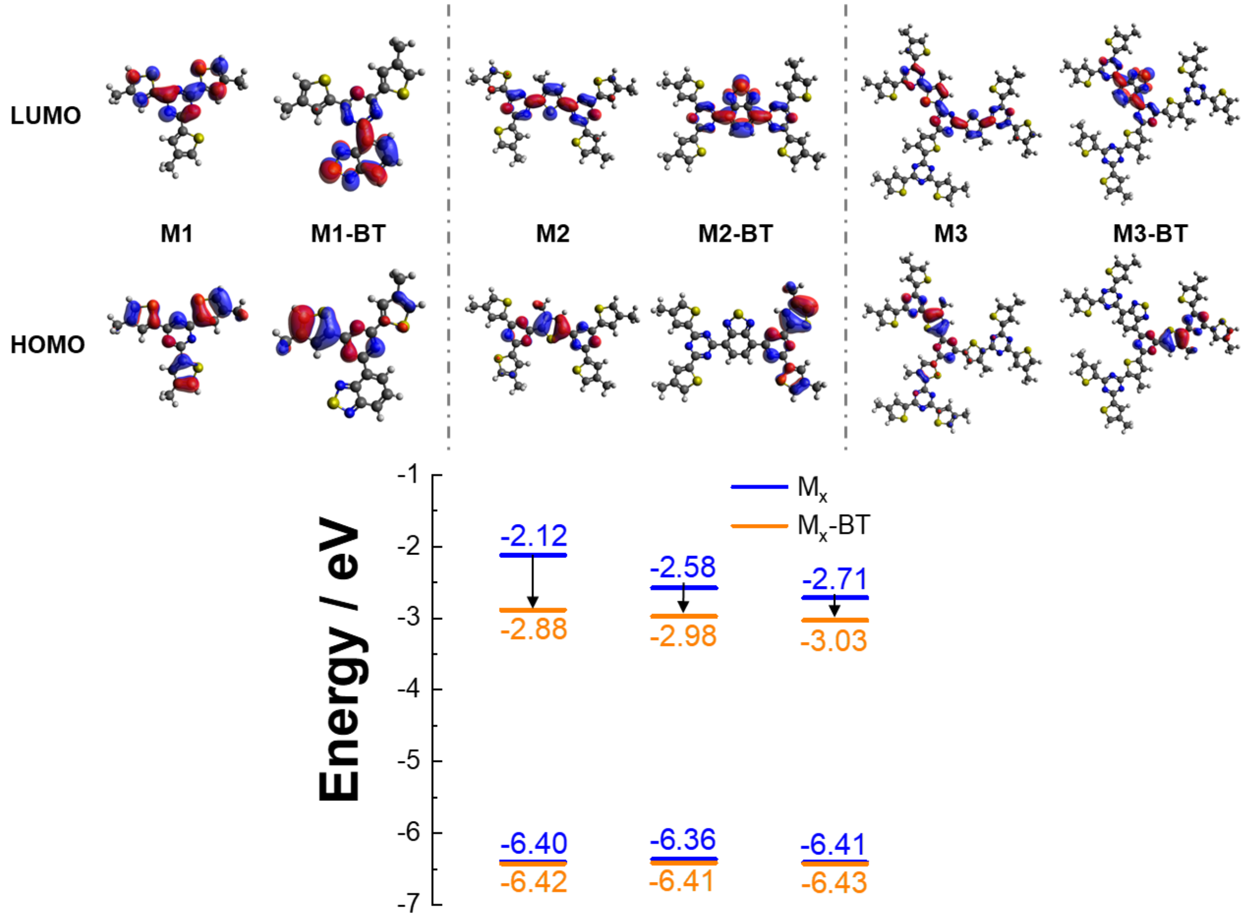

Figure S13. DFT Calculations on the B3LYP/6-31G(d) level for triazine structures analogous to the CTFs in this study. The structures were reduced to smaller units to limit the complexity of the theoretical calculations and were conducted to prove the general trend on frontier orbital energies upon benzothiadiazole incorporation.

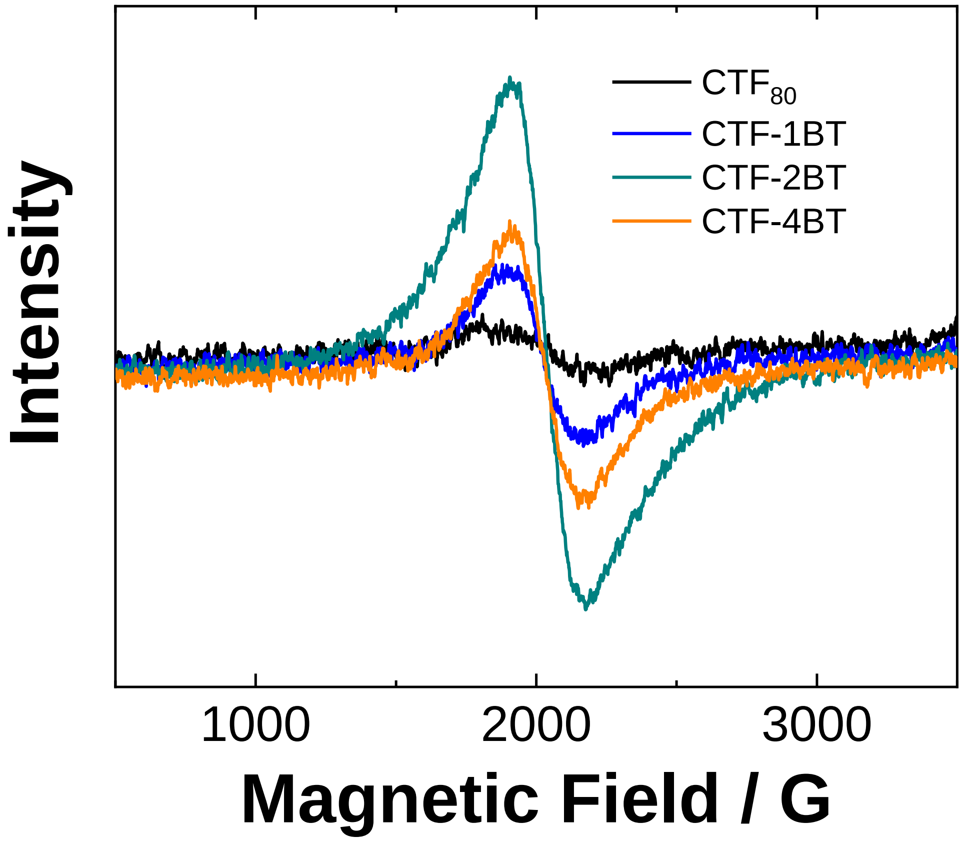

Figure S14. Electron paramagnetic resonance (EPR) spectra at room temperature with integrated blue light irradiation.

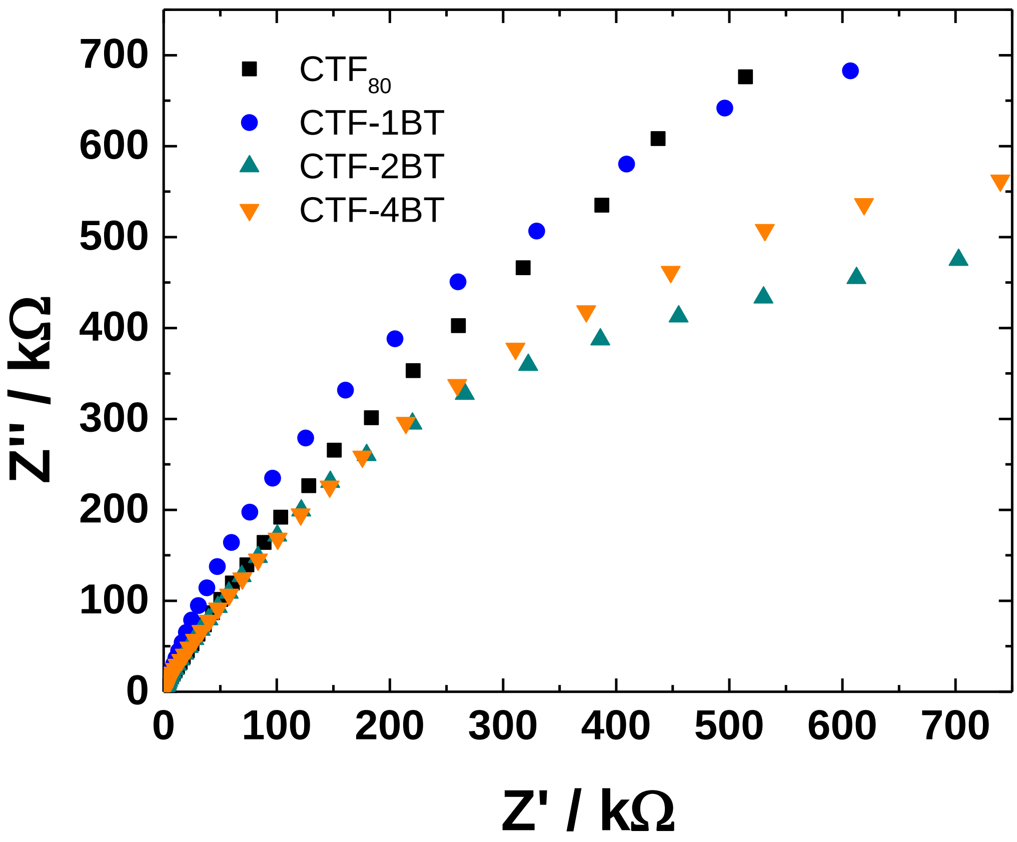

Figure S15. Impedance measurements of CTF NPs.

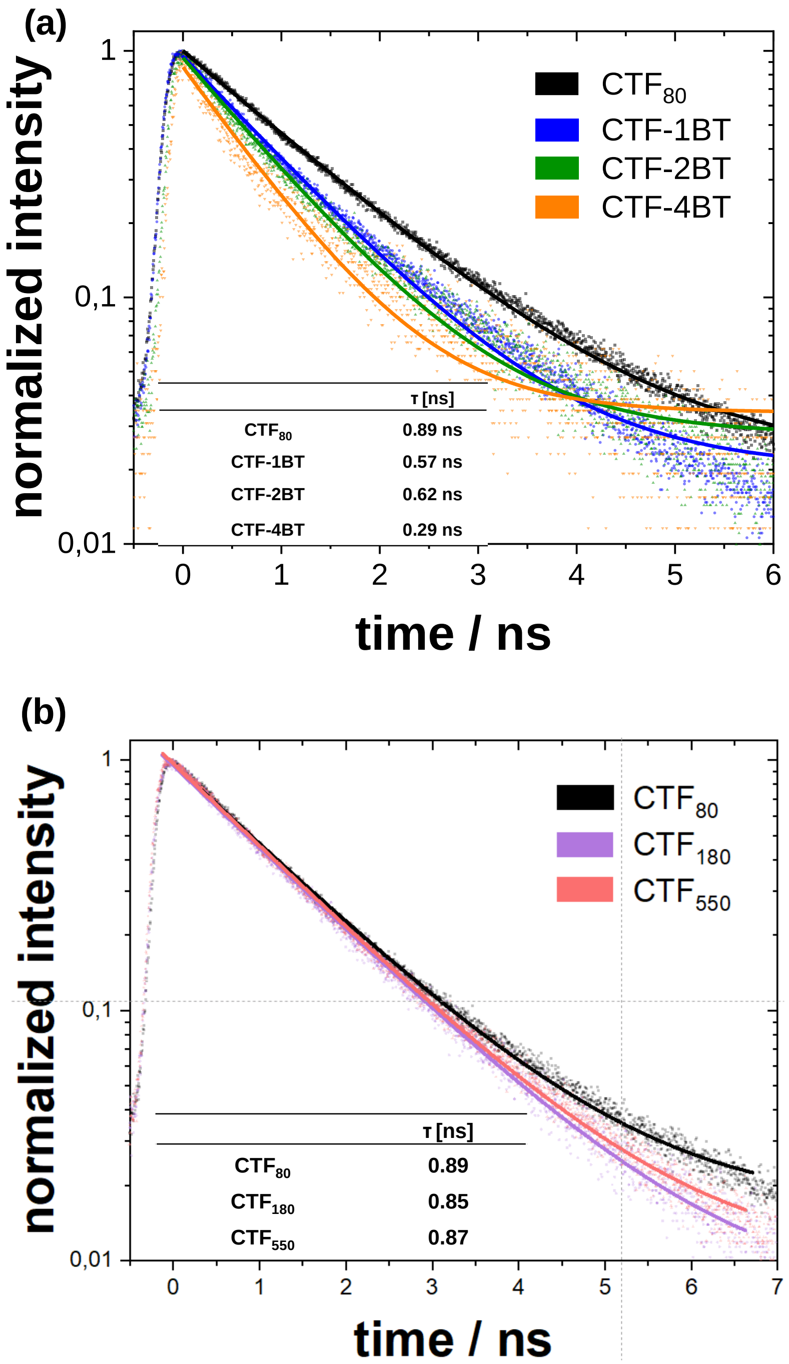

Figure S16. Time-resolved photoluminescence (TRPL) spectra of the CTF NPs at an excitation wavelength at 400 nm. (a) Effect of the BT content and (b) Size effect of the pristine CTF NPs.

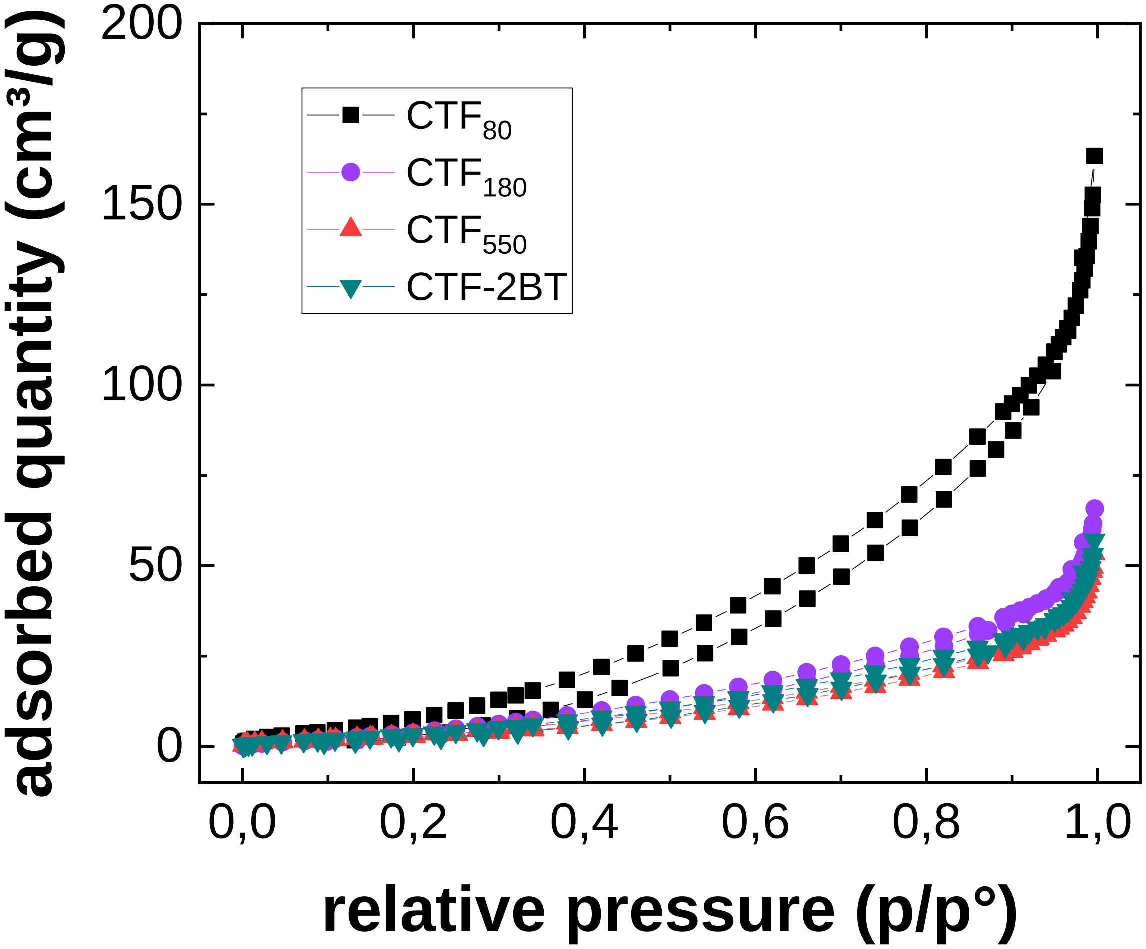

Figure S17. Nitrogen sorption isotherms for CTF NPs.

The nitrogen sorption isotherms are typical for non-porous materials. It is supposed, that porosity, if present, would not be detectable due to the long alkyl chains of DCHT blocking N_2 from accessing pores.

Table S1. Control reactions of the benchmark reaction. 4-methoxyphenol (0.1 mmol), anethole (0.15 mmol), $(NH_4)_2S_2O_8$ (0.2 mmol) and photocatalyst (2 mg) were added into solvent (2 mL). The mixture was irradiated with a blue LED lamp (*λ*=460 nm) for 22 h. Yield was determined by GC-MS with trimethylsilican as internal standard.

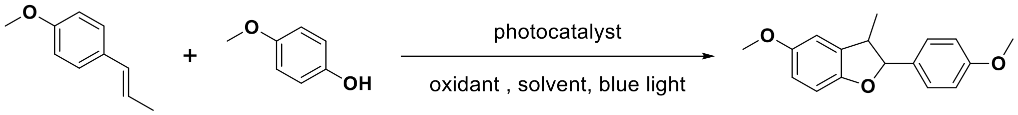

Entry	PC	Solvent	Oxidant	Time (h)	Conv. (%)
1	CTF_{80}	CH_3CN	Air (O_2)	22	40*
2	CTF_{80}	DMF	$(NH_4)_2S_2O_8$	22	<1
3	CTF_{80}	CH_3CN	$(NH_4)_2S_2O_8$	22	41
4	CTF_{80}	DMSO	$(NH_4)_2S_2O_8$	22	0
5	CTF_{80}	DCM	$(NH_4)_2S_2O_8$	22	51
6	CTF_{80}	DCE	$(NH_4)_2S_2O_8$	22	22
7	CTF_{80}	CH_3NO_2	$(NH_4)_2S_2O_8$	22	76
8	Bulk CTF	CH_3NO_2	$(NH_4)_2S_2O_8$	22	9
9	CTF - 1BT	CH_3NO_2	$(NH_4)_2S_2O_8$	22	87
10	CTF - 2BT	CH_3NO_2	$(NH_4)_2S_2O_8$	22	97
11	CTF - 4BT	CH_3NO_2	$(NH_4)_2S_2O_8$	22	89

Entry	PC	Light	Oxidant	Scavengers	Time (h)	Conv. (%)
1	-	+	+	-	22	3
2	+	-	+	-	22	<1
3	+	+	-		22	9
4	+	+	+	$CuCl_2$	22	95
5	+	+	+	KI	22	2

*****In entry 1 with O_2 from air as oxidant, multiple products (>4) can be detected by GC-MS. The desired compound is among the products but could not be synthesized selectively.

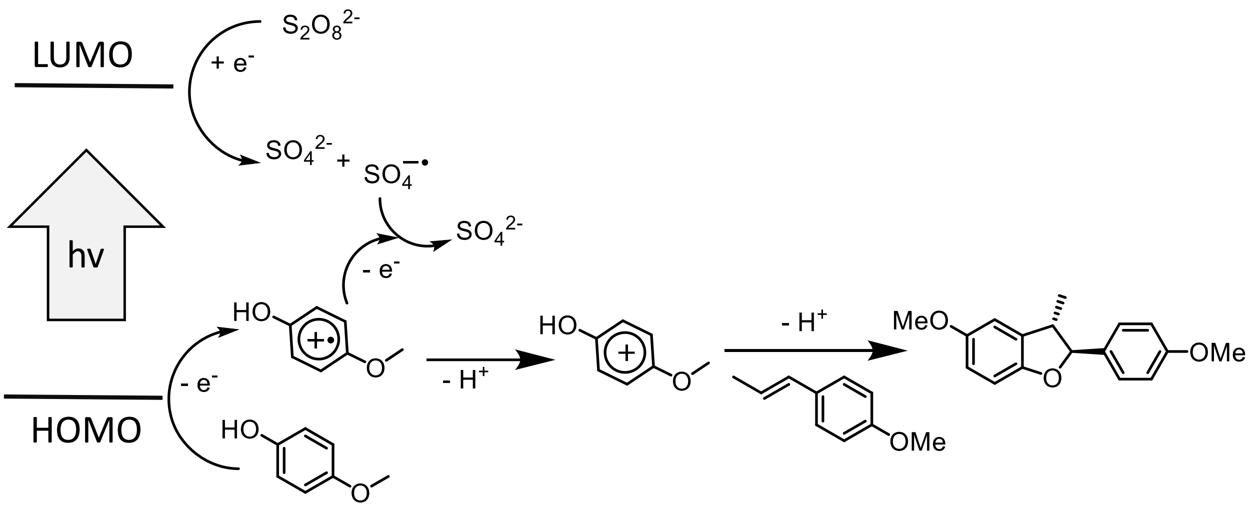

Figure S18. Proposed mechanism of the oxidative [3+2] cycloaddition catalysed by the CTF NPs.

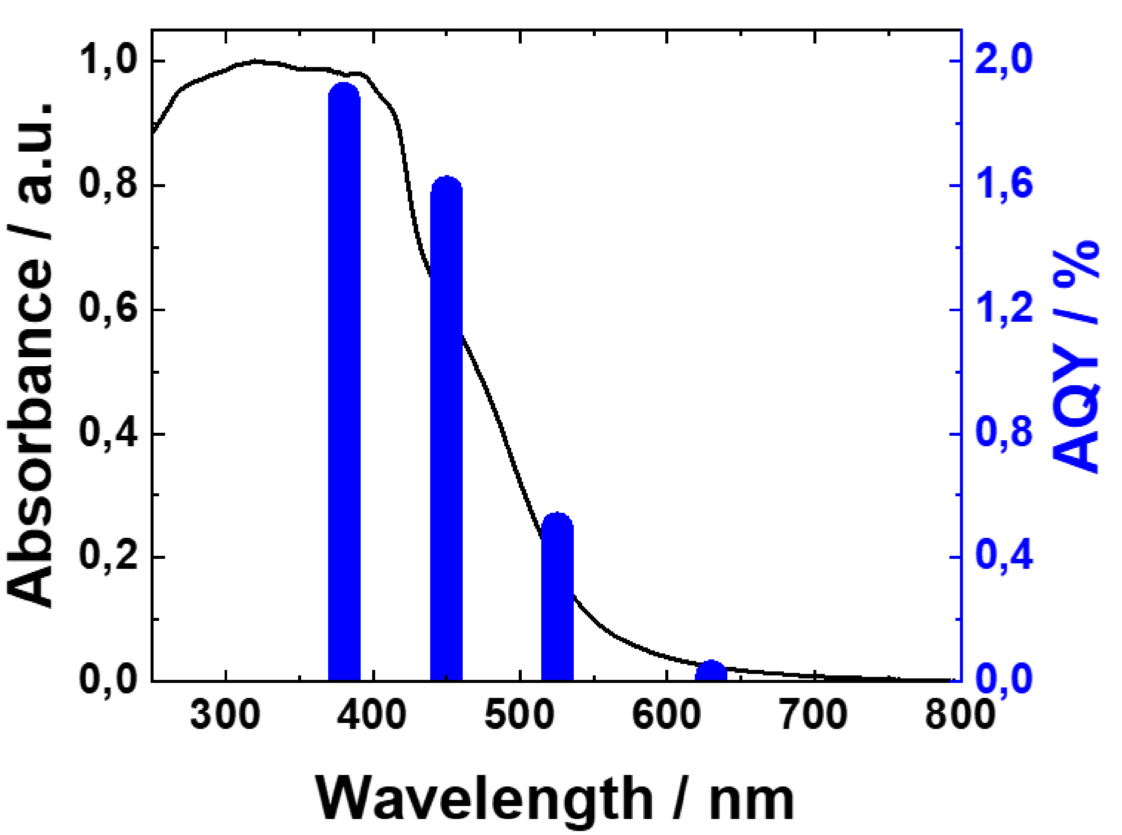

Figure S19. Apparent quantum yield (AQY) for CTF-2BT when irradiated at four different wavelengths.

For determining the apparent quantum yield, CTF-2BT was used as photocatalyst. The general procedure of the photocatalytic dibenzofurane benchmark synthesis was followed. The product formation was studied using four different LEDs (385 nm, 450 nm, 525 nm, 620 nm, ~60 mW/cm²). An area of 1.44 cm² was illuminated for 4h. The apparent quantum yields were estimated using the following equation

$$\Phi_{\text{AQY}} = \frac{\text{number of product molecules formed}}{\text{number of incident photons}}$$

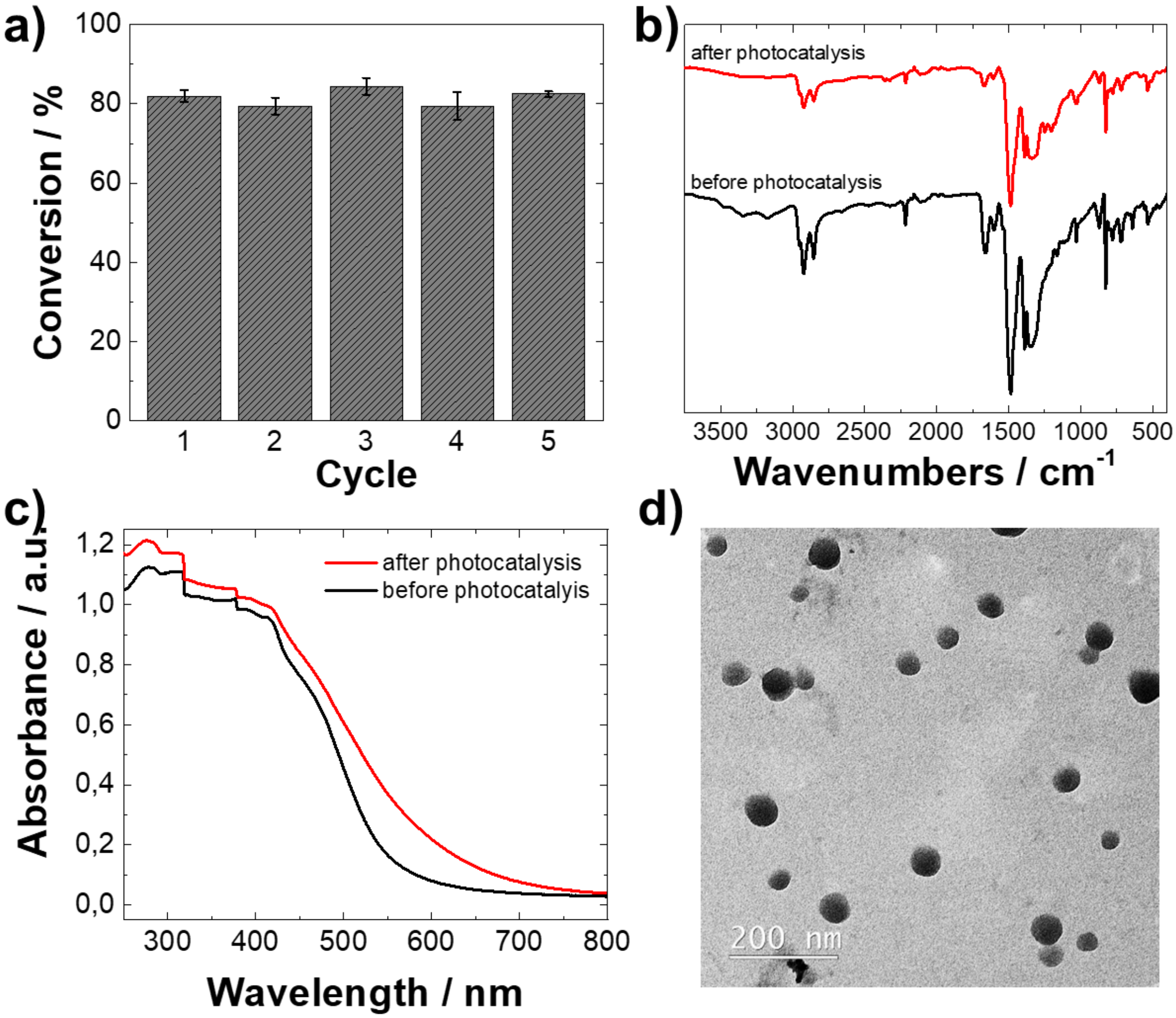

Figure S20. Bar diagram of yields after 24h of reaction using CTF_{80} as photocatalyst in the benchmark reaction. The error bar represents two independent measurements. UV/Vis and IR spectra before and after (b,c) as well as TEM image after the application (d).

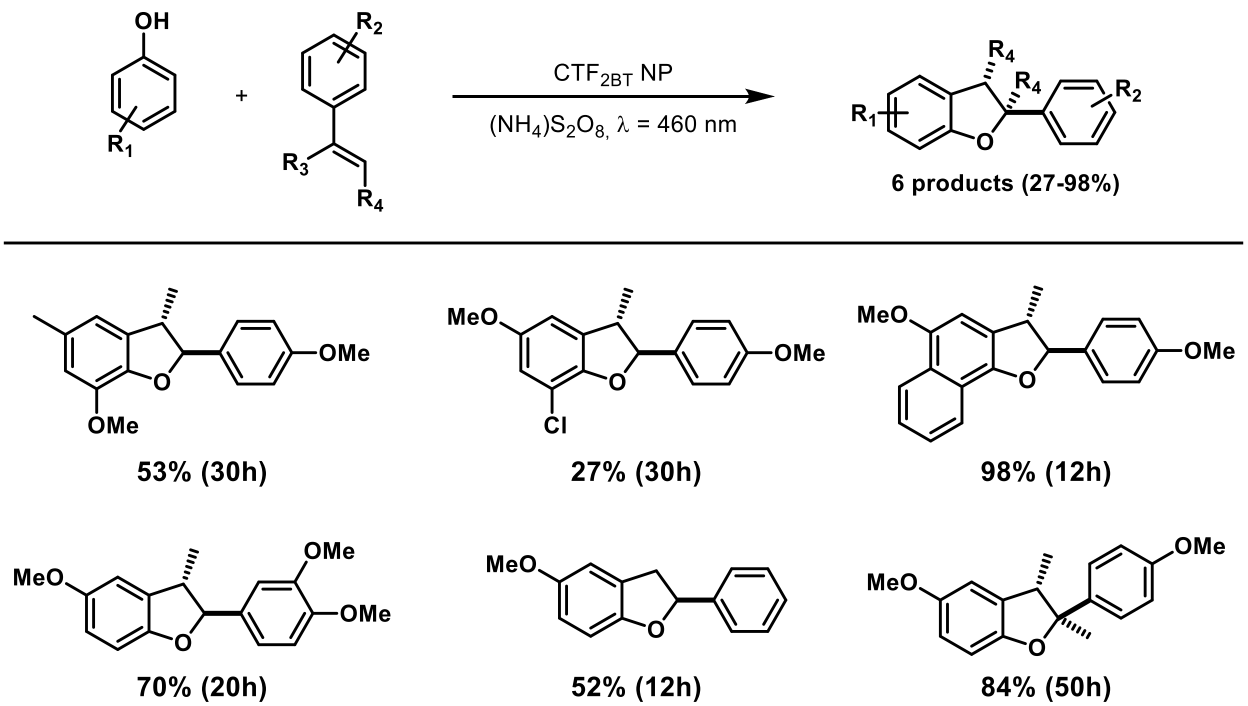

Figure S21. Overview of the substrate scope for different 2,3-dibenzofurans catalysed using CTF-2BT NPs. Experimental detail is given in the general procedure.

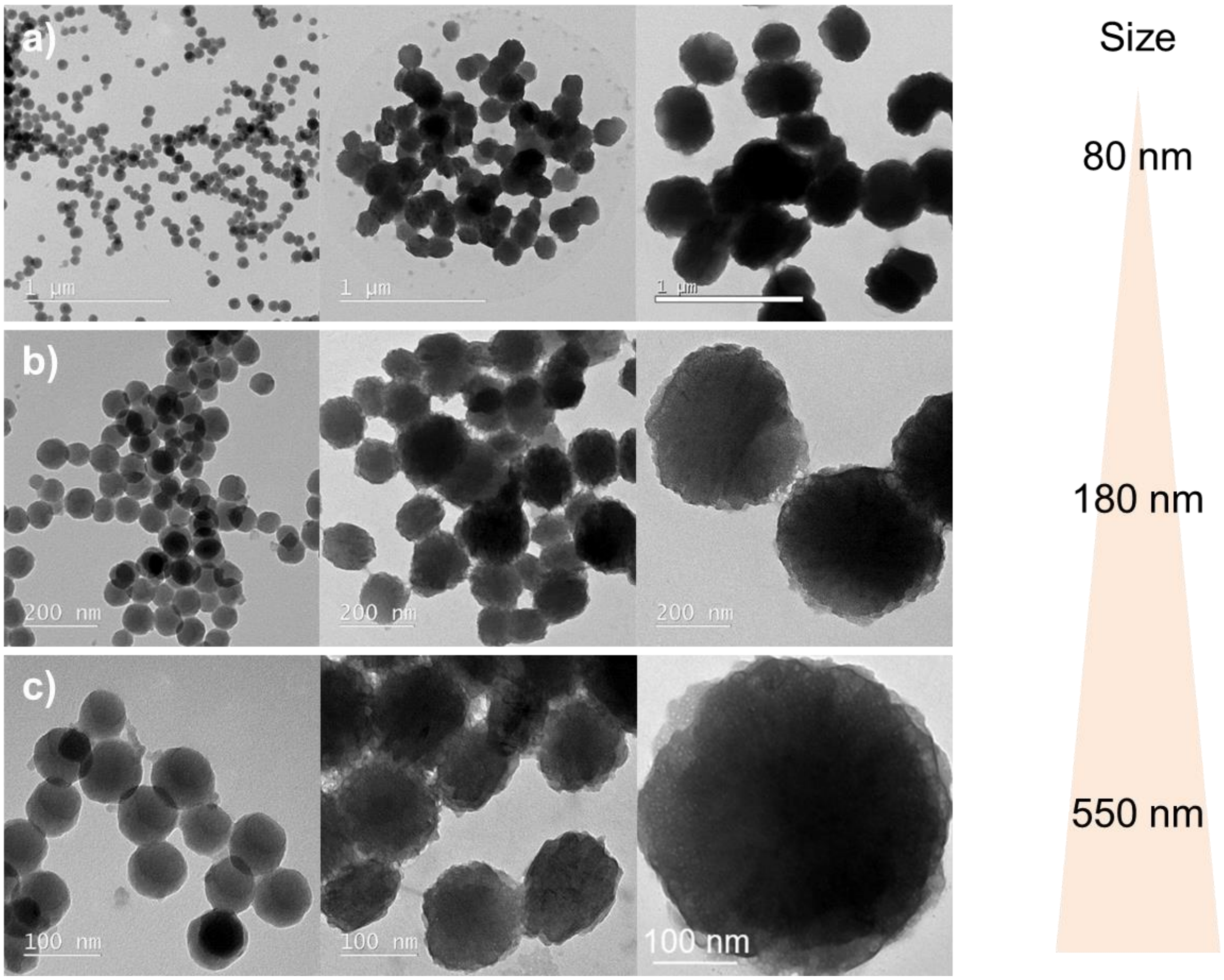

Figure S22. Transmission electron microscopy of differently sized CTF-NPs.

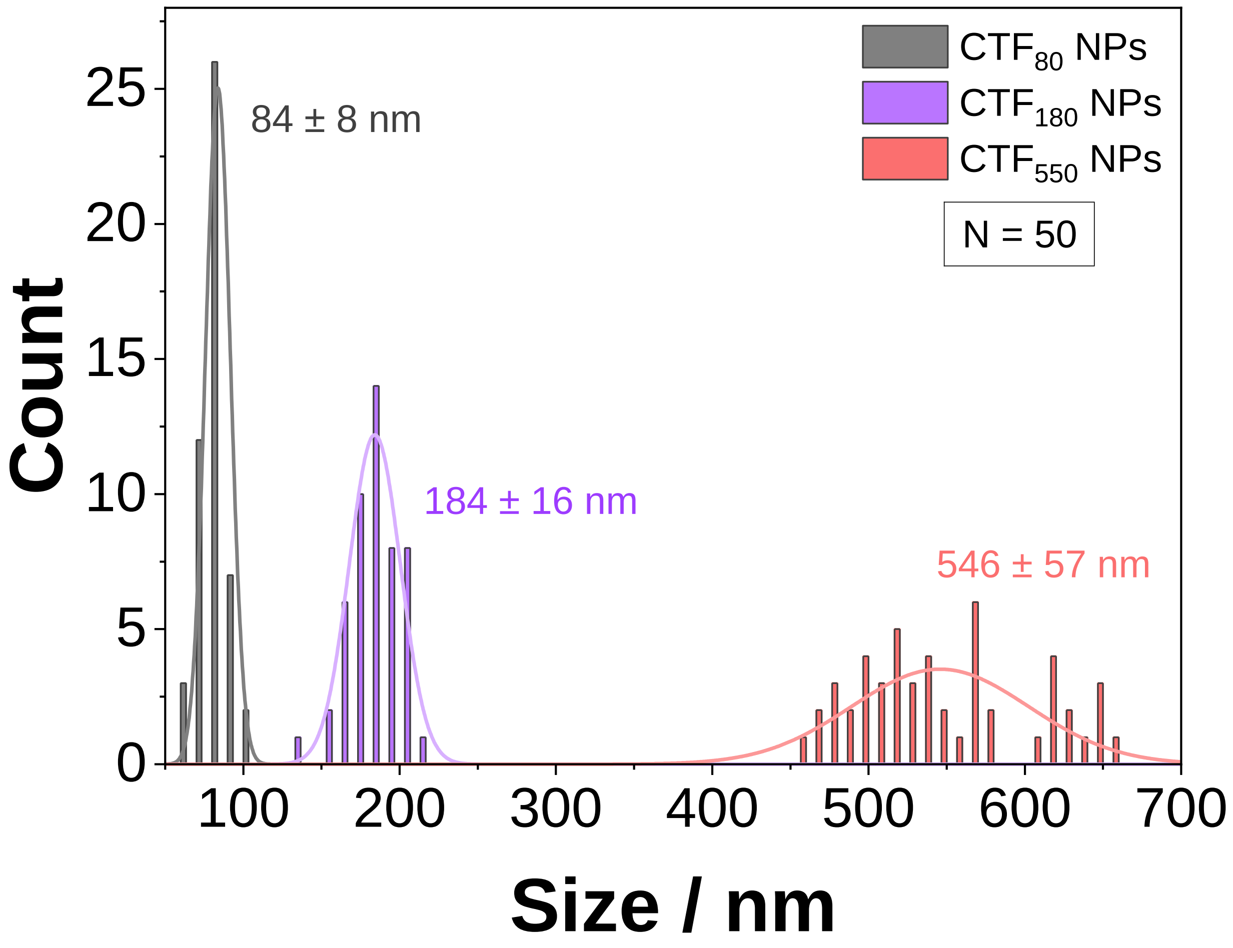

Figure S23. Size histogram of counted nanoparticles (N=50) from transmission electron microscopy images.

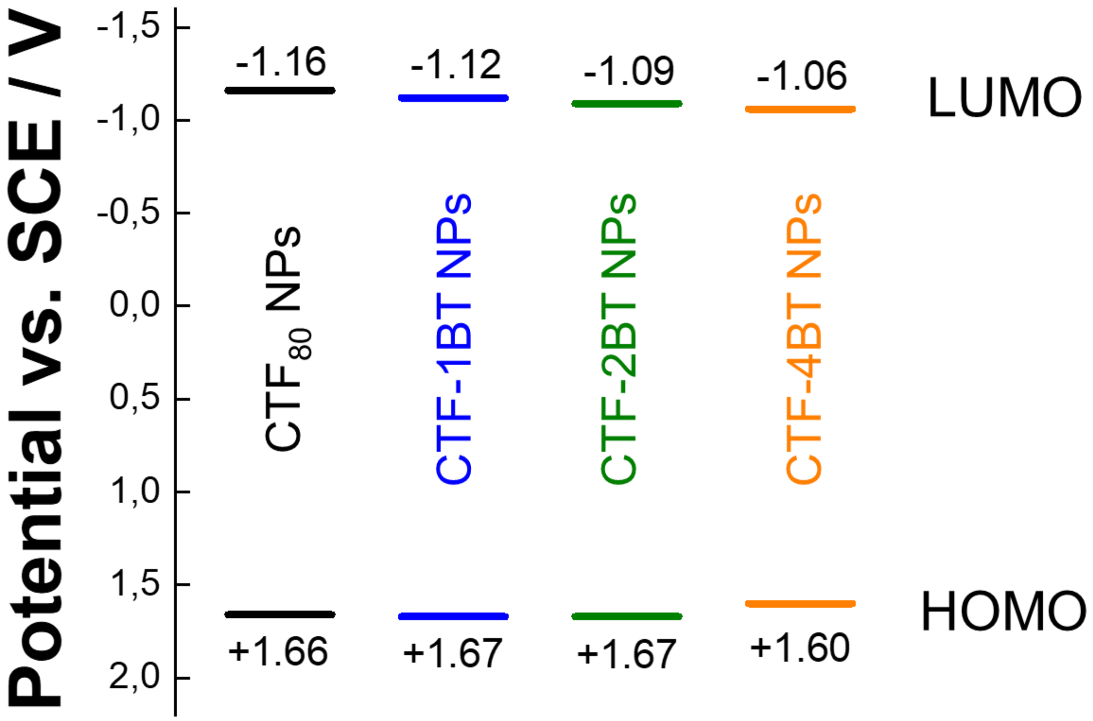

Figure S24. Comparison of HOMO and LUMO positions for CTF-NPs with different BT content**.** LUMO values result from CV measurements; HOMO levels are deducted combining CV values and bandgaps from the Kubelka-Munk-transformed diffuse reflectance UV/Vis spectra.

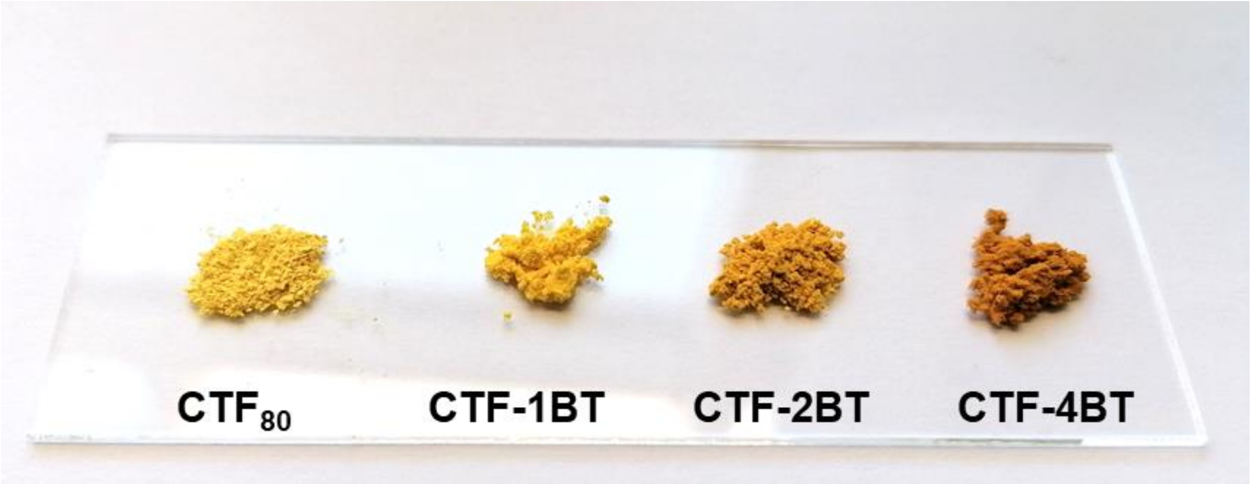

Figure S15. Photograph of the CTF nanoparticles with different contents of BT.

NMR spectra of the products

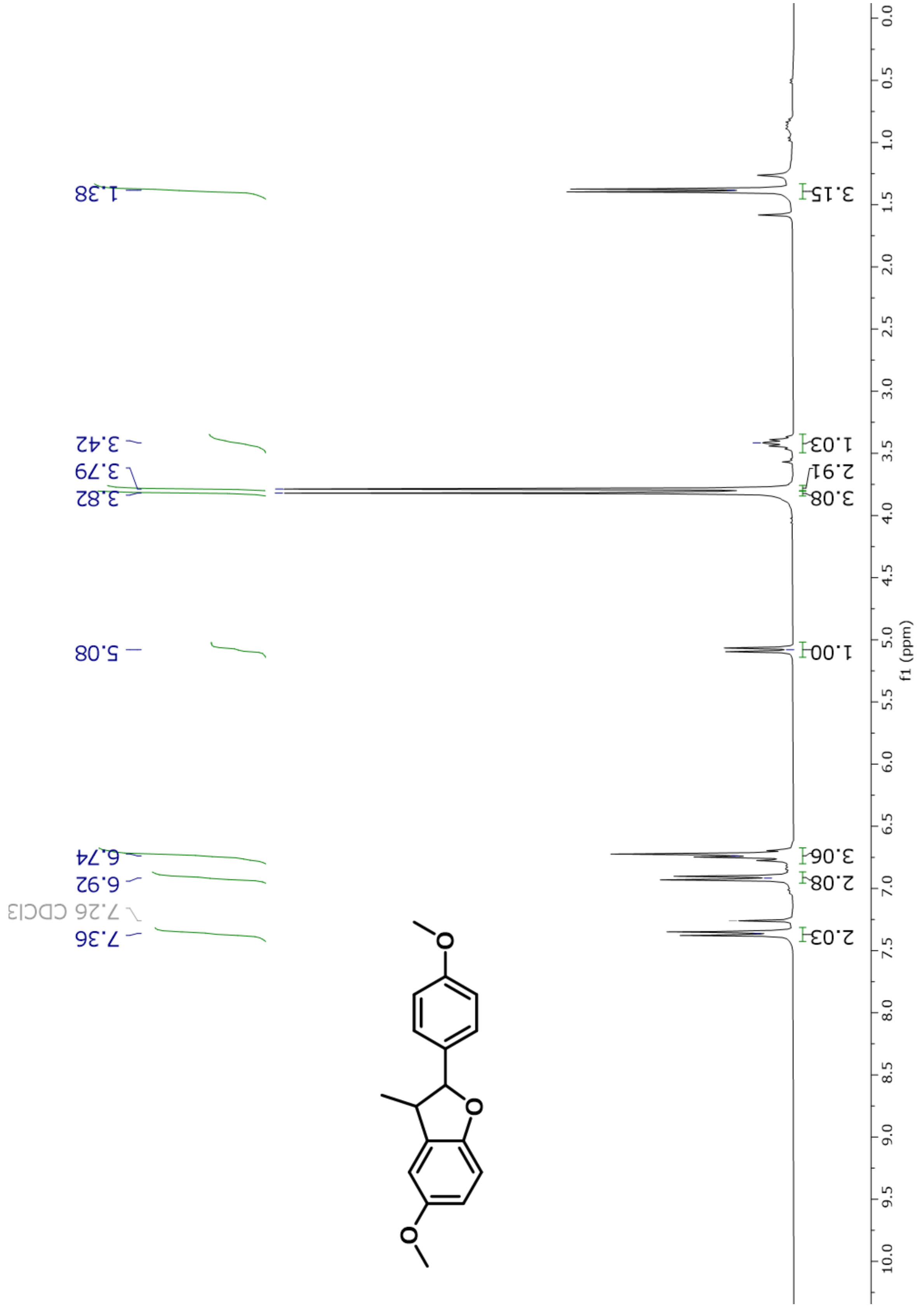

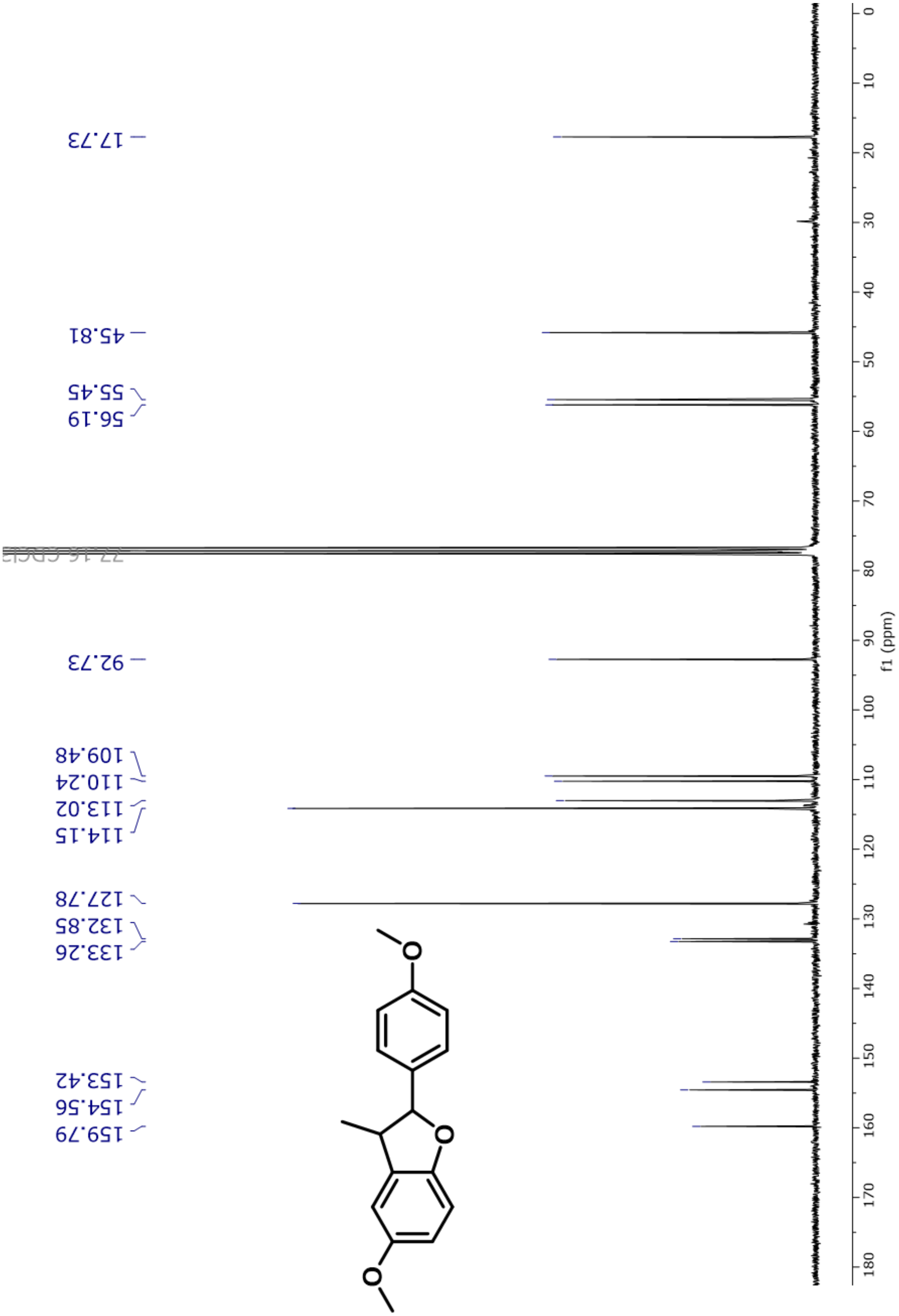
17.73
45.81
55.45
56.19
77.16 CDCl3
92.73
109.48
110.24
113.02
114.15
127.78
132.85
133.26
153.42
154.56
159.79
f1 (ppm)

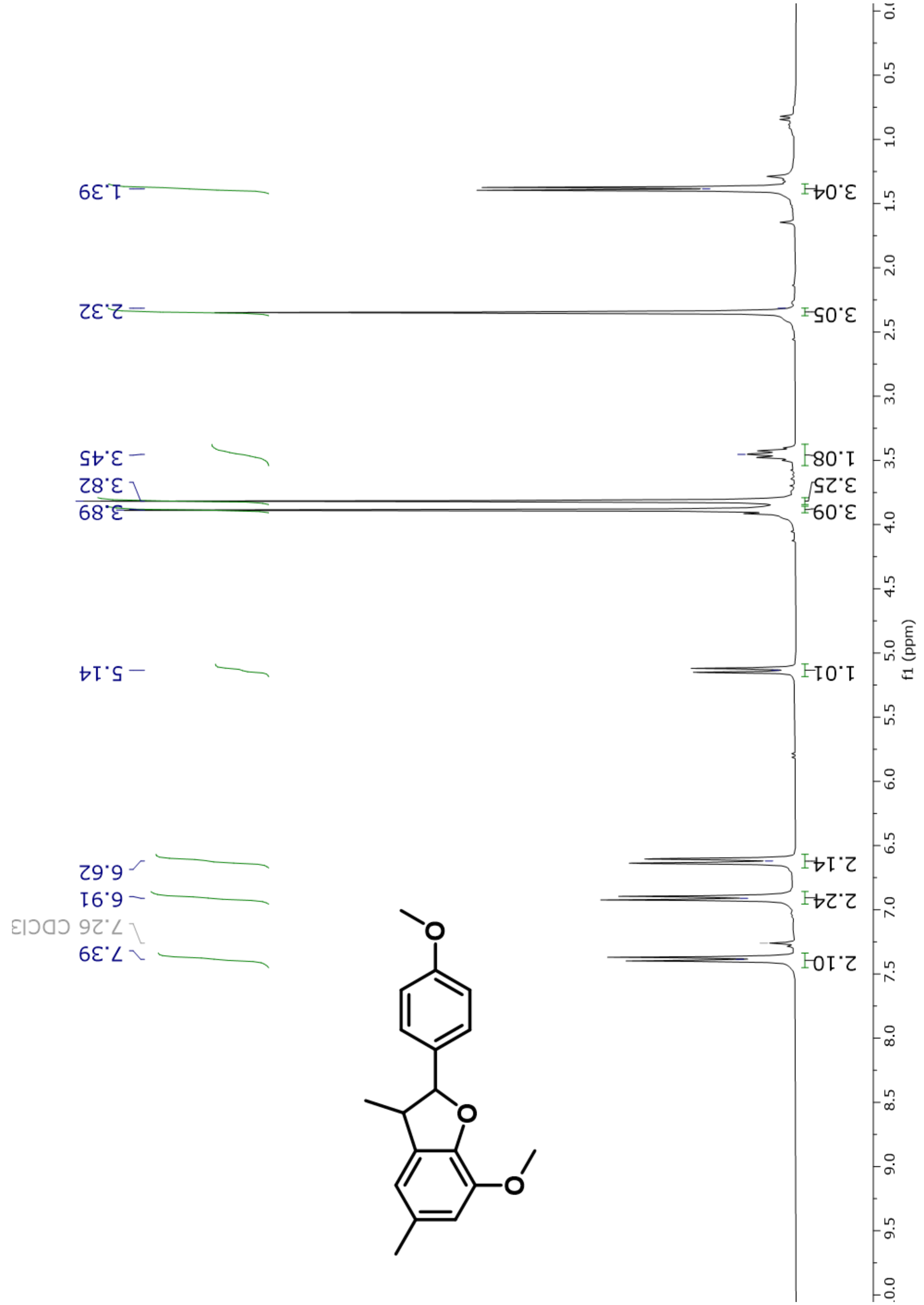

1.39
2.32
3.45
3.82
3.89
5.14
6.62
6.91
7.26 CDCl3
7.39
3.04
3.05
1.08
3.25
3.09
1.01
2.14
2.24
2.10
f1 (ppm)

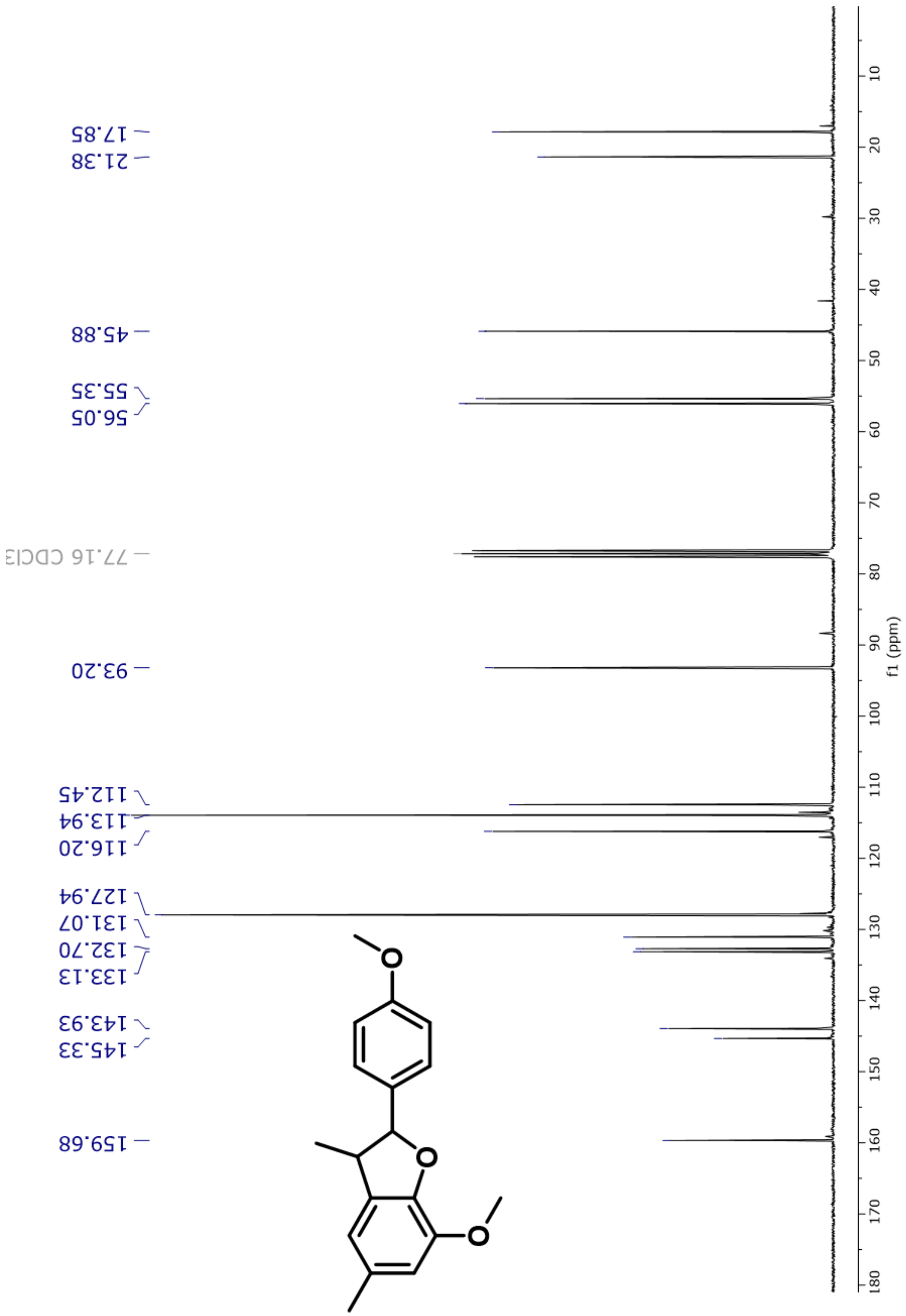
17.85
21.38
45.88
55.35
56.05
77.16 CDCl3
93.20
112.45
113.94
116.20
127.94
131.07
132.70
133.13
143.93
145.33
159.68
f1 (ppm)

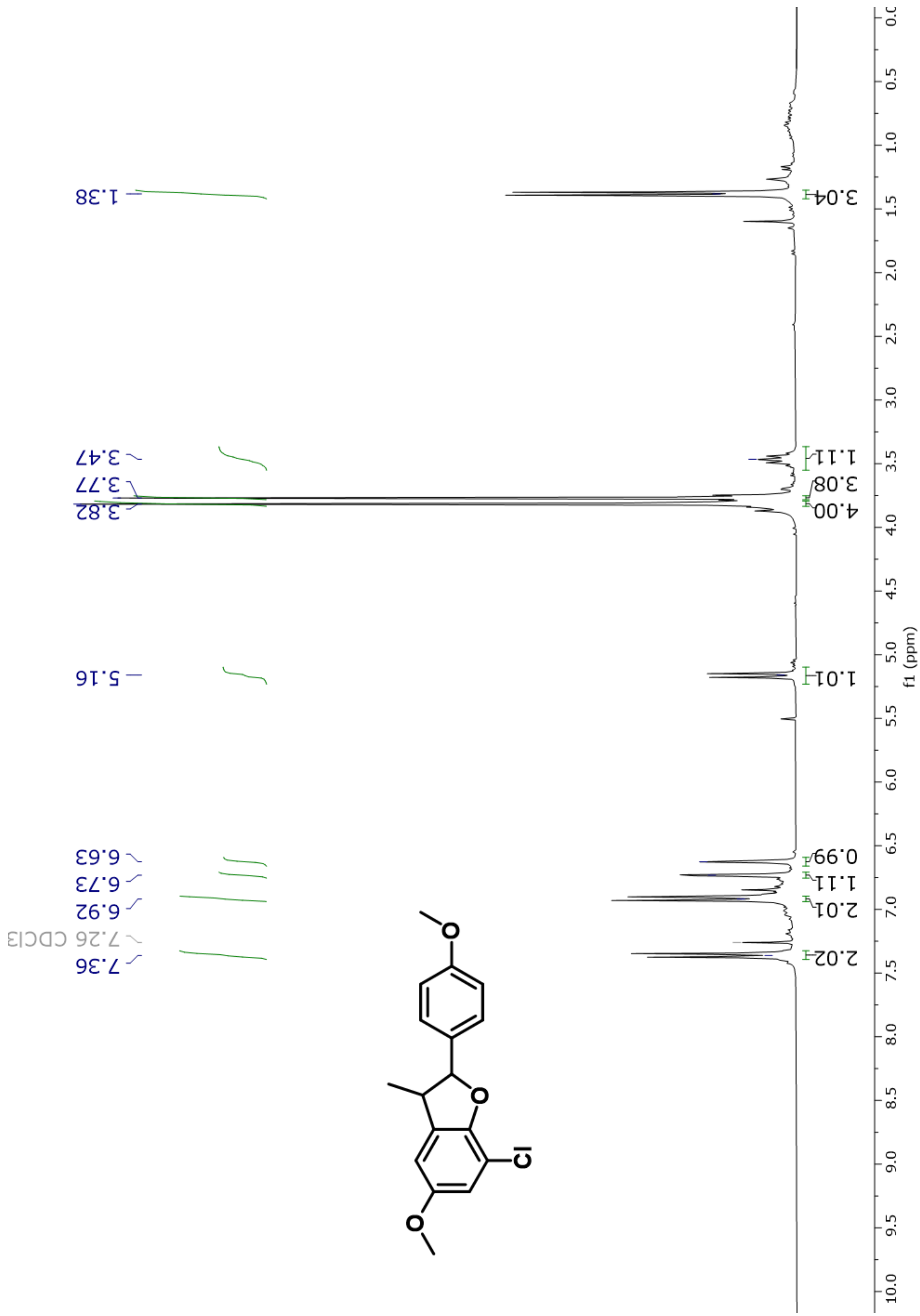

1.38
3.47
3.77
3.82
5.16
6.63
6.73
6.92
7.26 CDCl3
7.36
3.04
1.11
3.08
4.00
1.01
0.99
1.11
2.01
2.02
f1 (ppm)
Cl

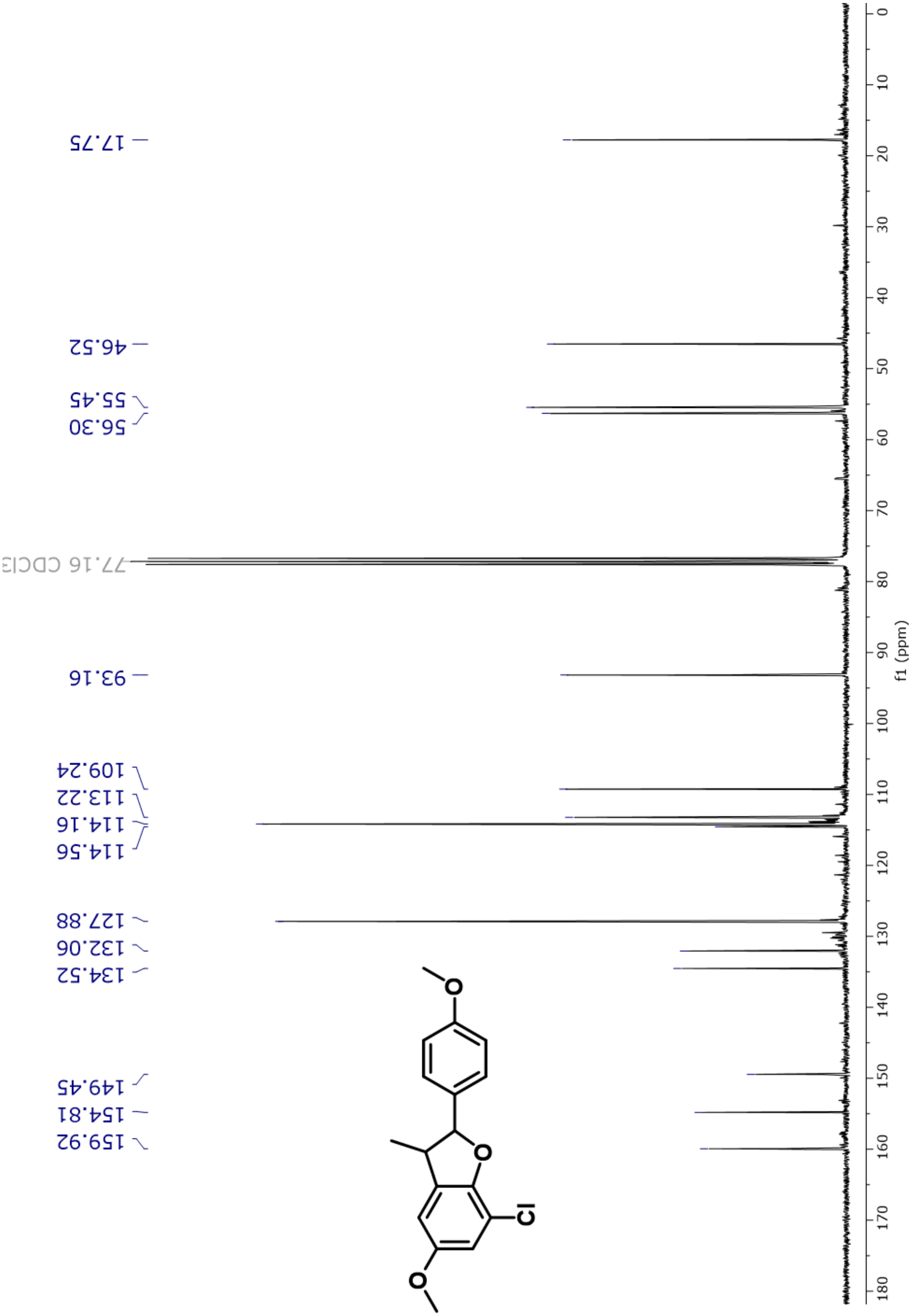
17.75
46.52
55.45
56.30
77.16 CDCl3
93.16
109.24
113.22
114.16
114.56
127.88
132.06
134.52
149.45
154.81
159.92
Cl
O
f1 (ppm)

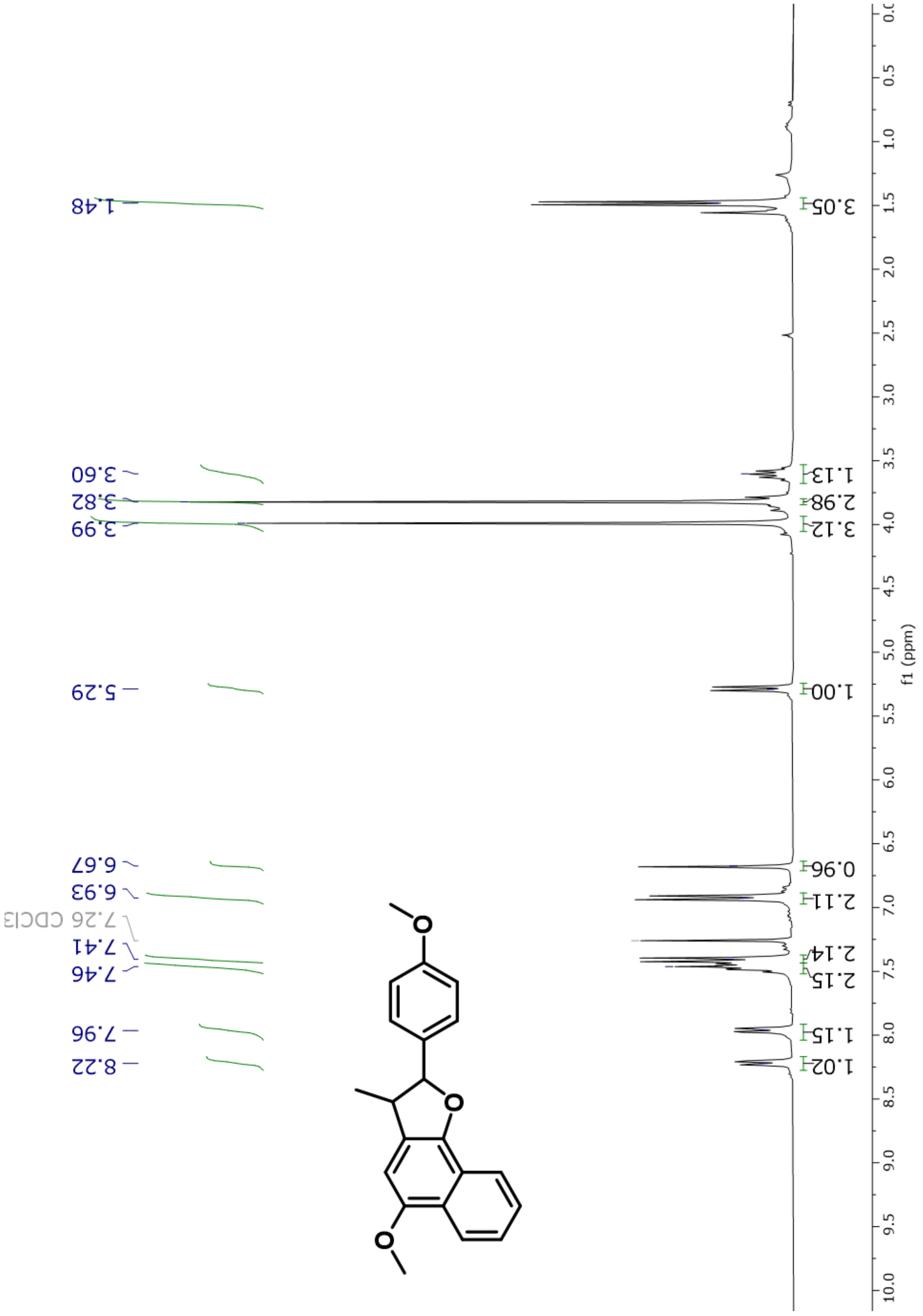

1.48
3.60
3.82
3.99
5.29
6.67
6.93
7.26 CDCl3
7.41
7.46
7.96
8.22
3.05
1.13
2.98
3.12
1.00
0.96
2.11
2.14
2.15
1.15
1.02
f1 (ppm)

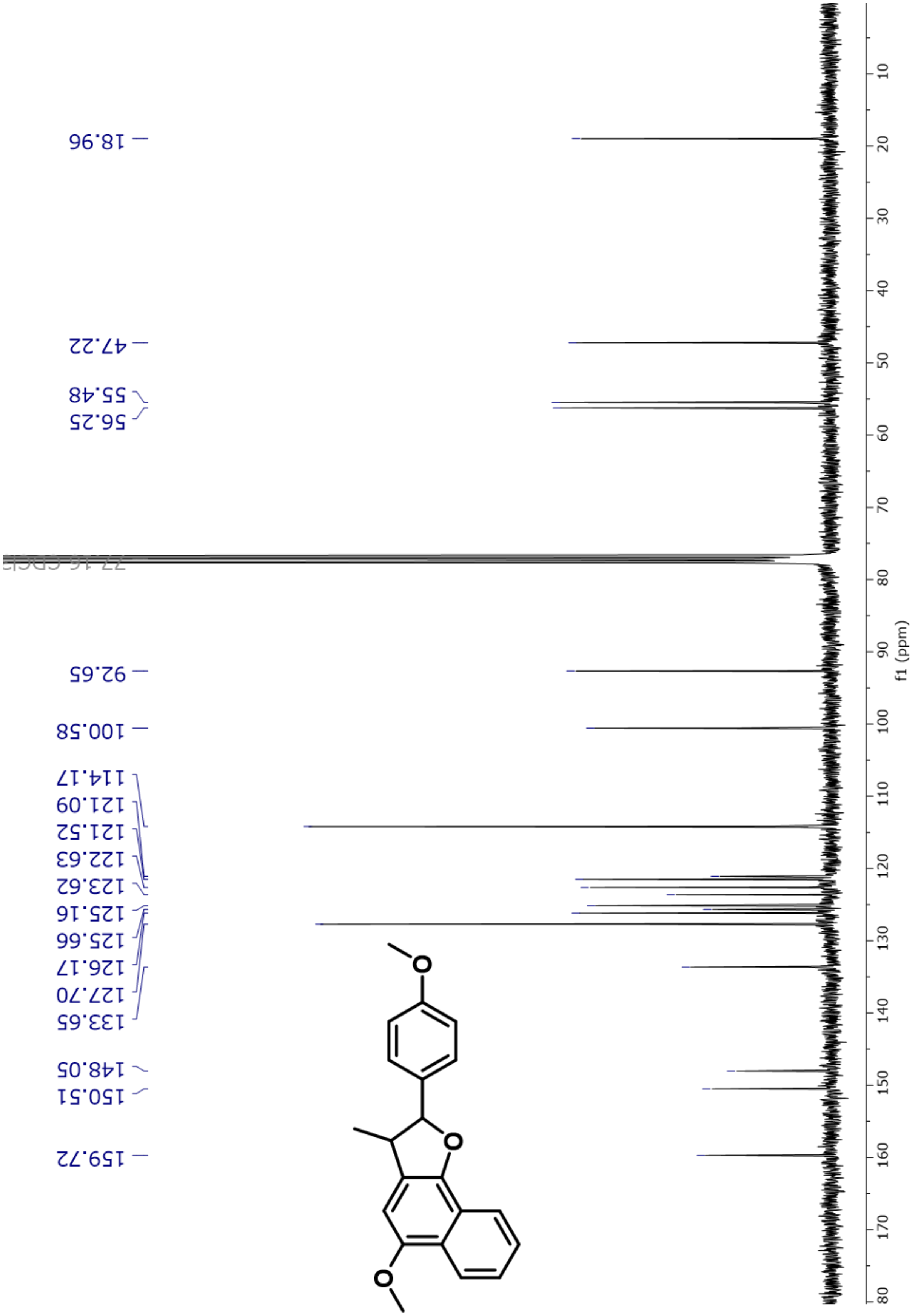
18.96
47.22
55.48
56.25
92.65
100.58
114.17
121.09
121.52
122.63
123.62
125.16
125.66
126.17
127.70
133.65
148.05
150.51
159.72
f1 (ppm)

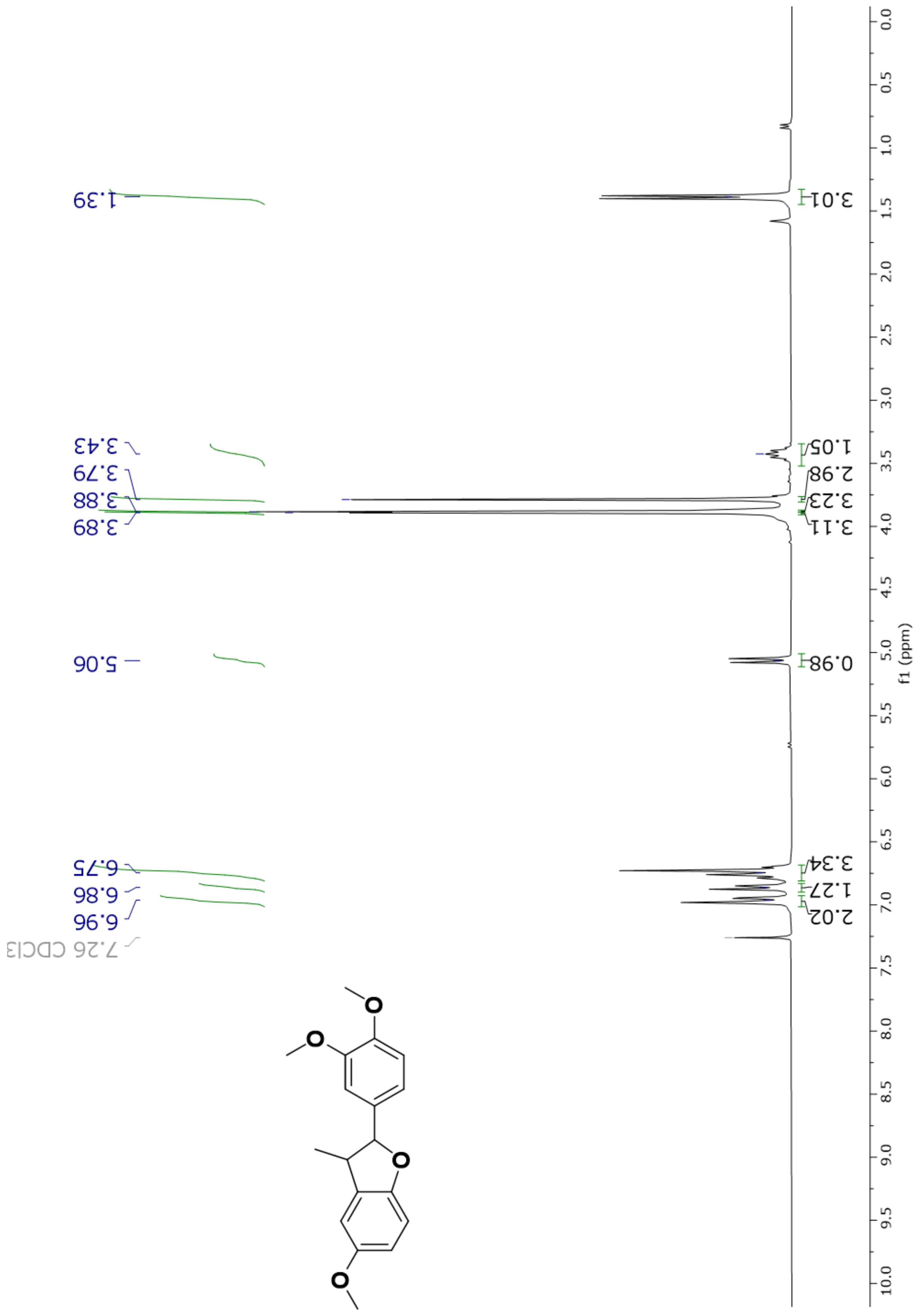
1.39
3.43
3.79
3.88
3.89
5.06
6.75
6.86
6.96
7.26 CDCl3
3.01
1.05
2.98
3.23
3.11
0.98
3.34
1.27
2.02
f1 (ppm)

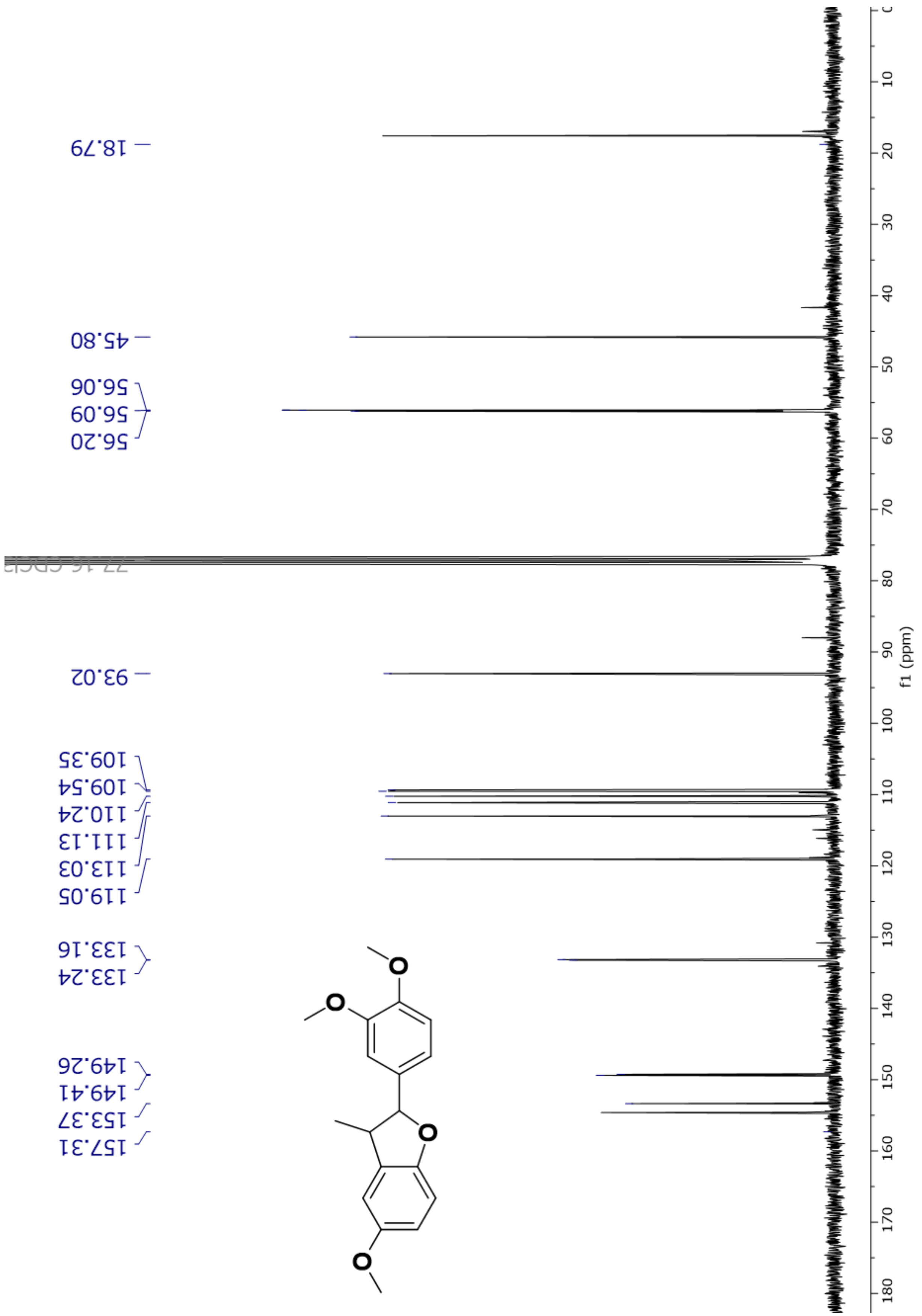

18.79
45.80
56.06
56.09
56.20
93.02
109.35
109.54
110.24
111.13
113.03
119.05
133.16
133.24
149.26
149.41
153.37
157.31
f1 (ppm)

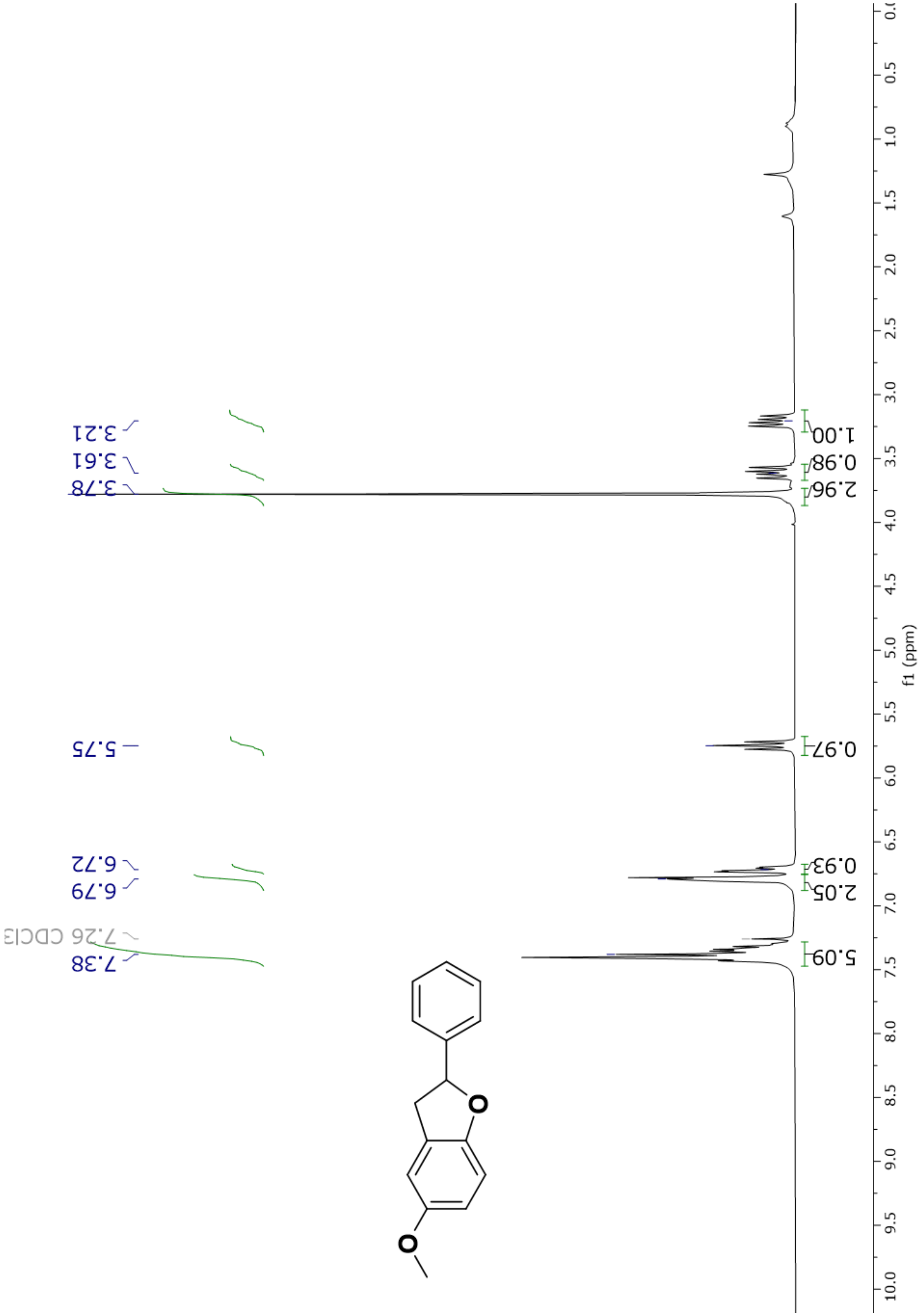
3.21
3.61
3.78
5.75
6.72
6.79
7.26 CDCl3
7.38
1.00
0.98
2.96
0.97
0.93
2.05
5.09
f1 (ppm)

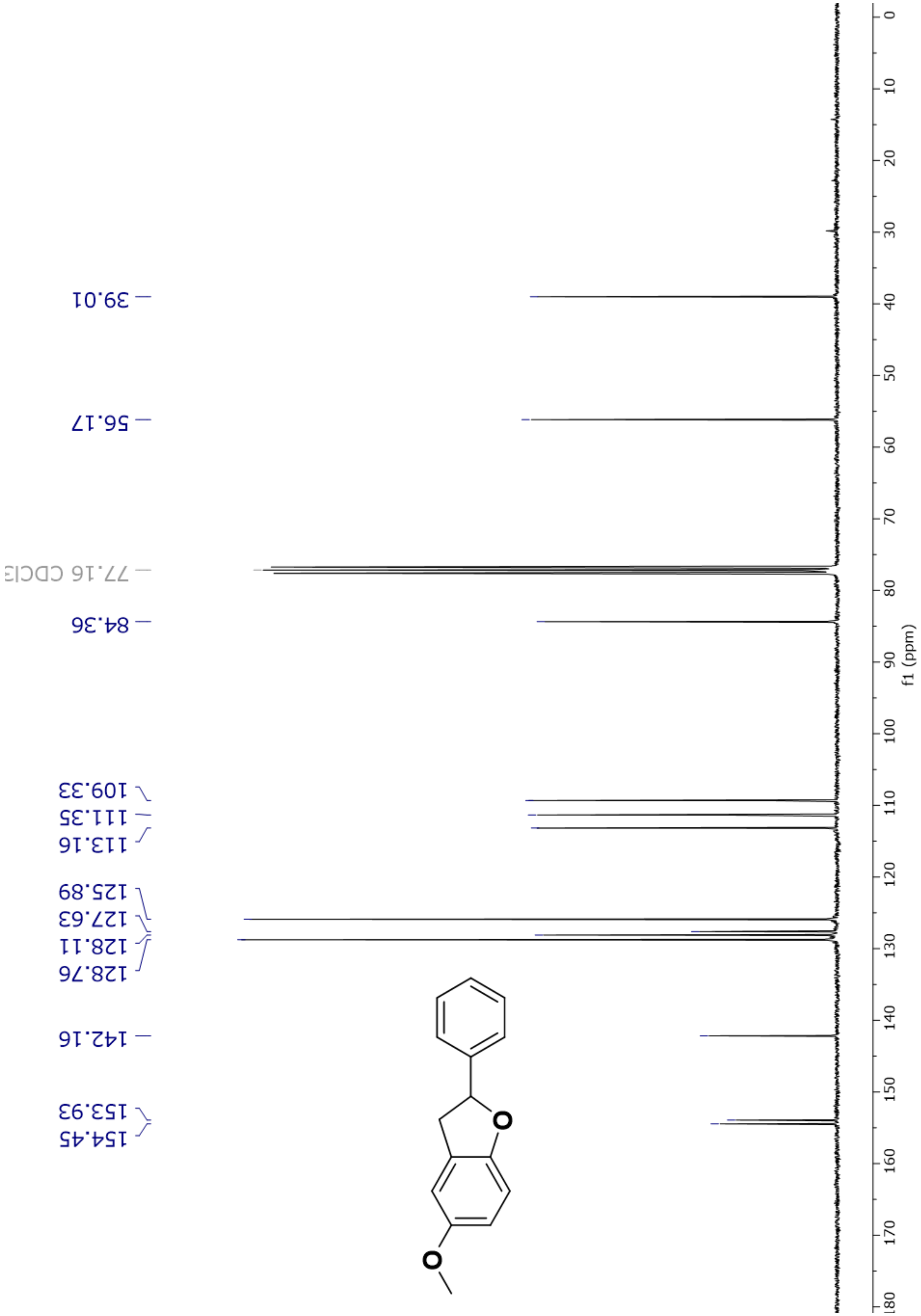

39.01
56.17
77.16 CDCl3
84.36
109.33
111.35
113.16
125.89
127.63
128.11
128.76
142.16
153.93
154.45
f1 (ppm)

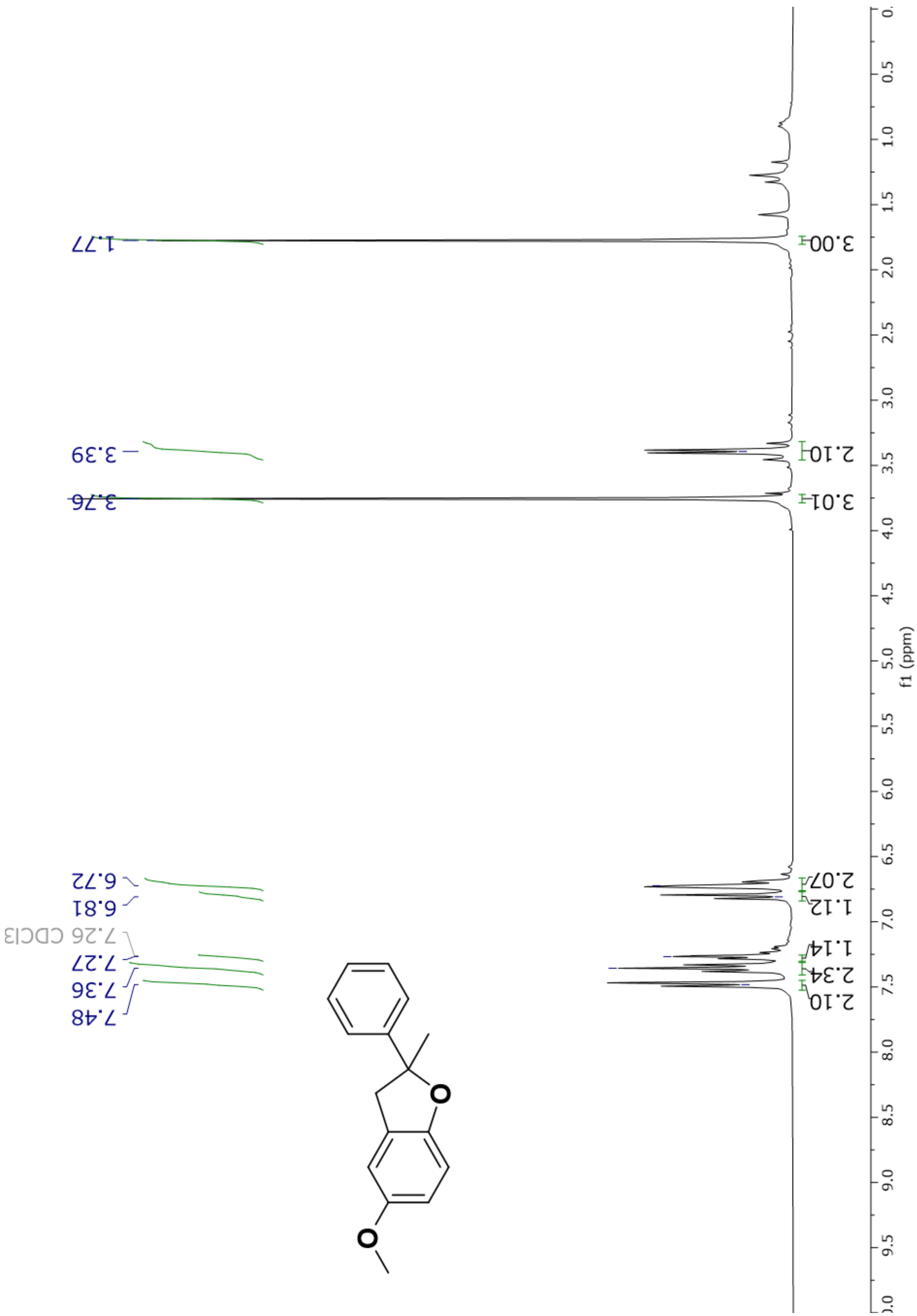

1.77
3.39
3.76
6.72
6.81
7.26 CDCl3
7.27
7.36
7.48
3.00
2.10
3.01
2.07
1.12
1.14
2.34
2.10
f1 (ppm)

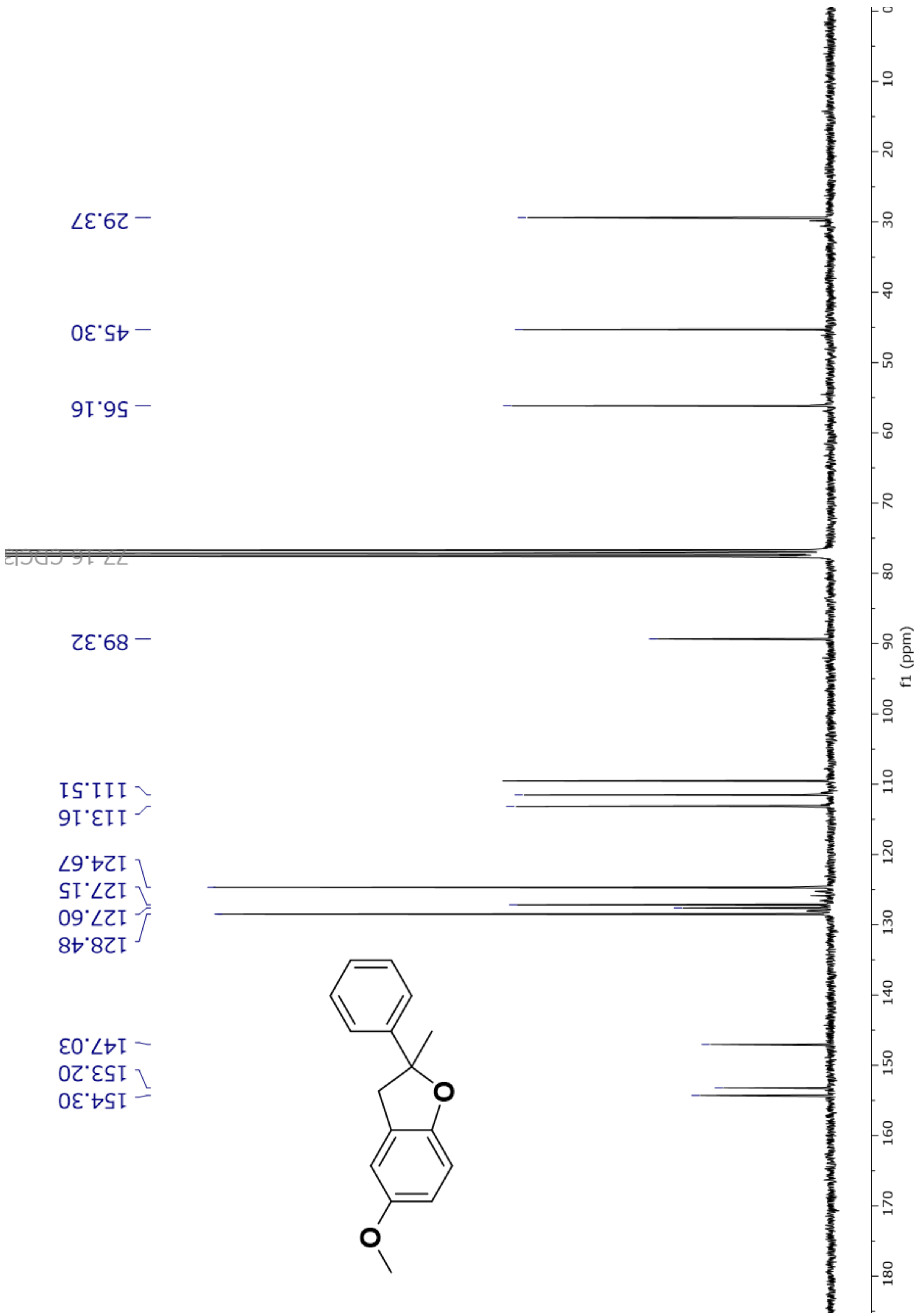
29.37
45.30
56.16
77.16 CDCl3
89.32
111.51
113.16
124.67
127.15
127.60
128.48
147.03
153.20
154.30
f1 (ppm)

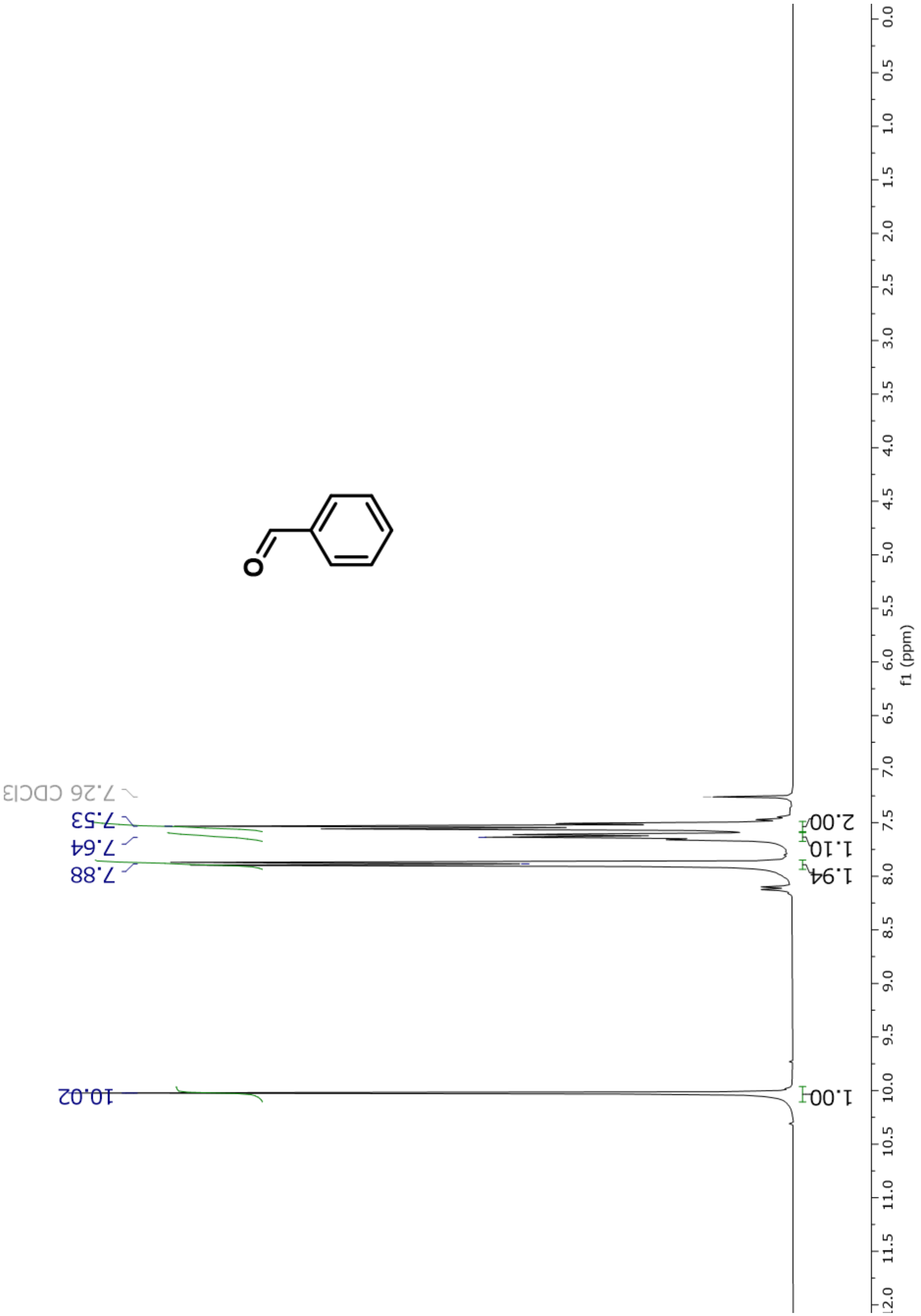
7.26 CDCl3
7.53
7.64
7.88
10.02
2.00
1.10
1.94
1.00
f1 (ppm)

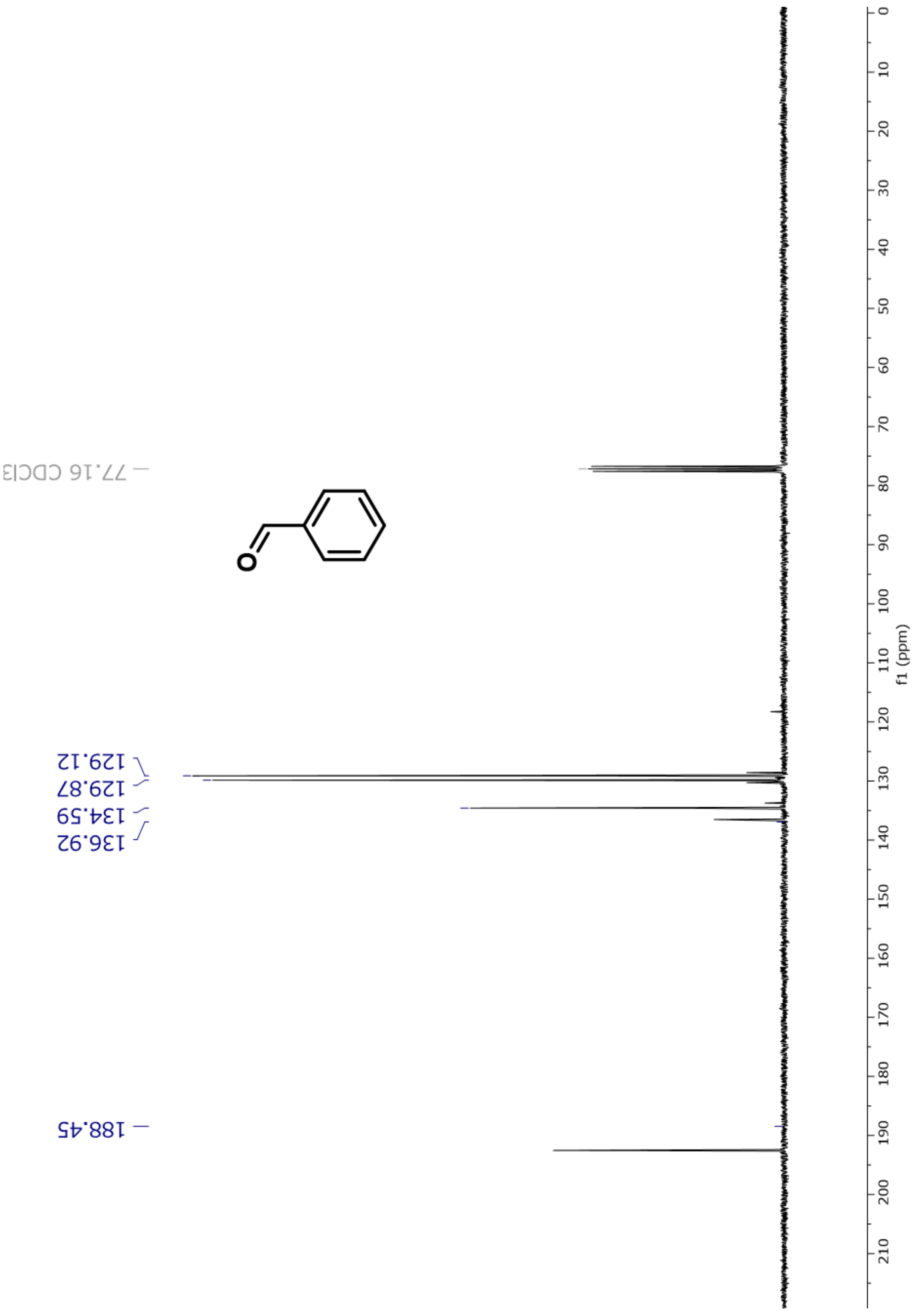

77.16 CDCl3
129.12
129.87
134.59
136.92
188.45
f1 (ppm)
0
10
20
30
40
50
60
70
80
90
100
110
120
130
140
150
160
170
180
190
200
210

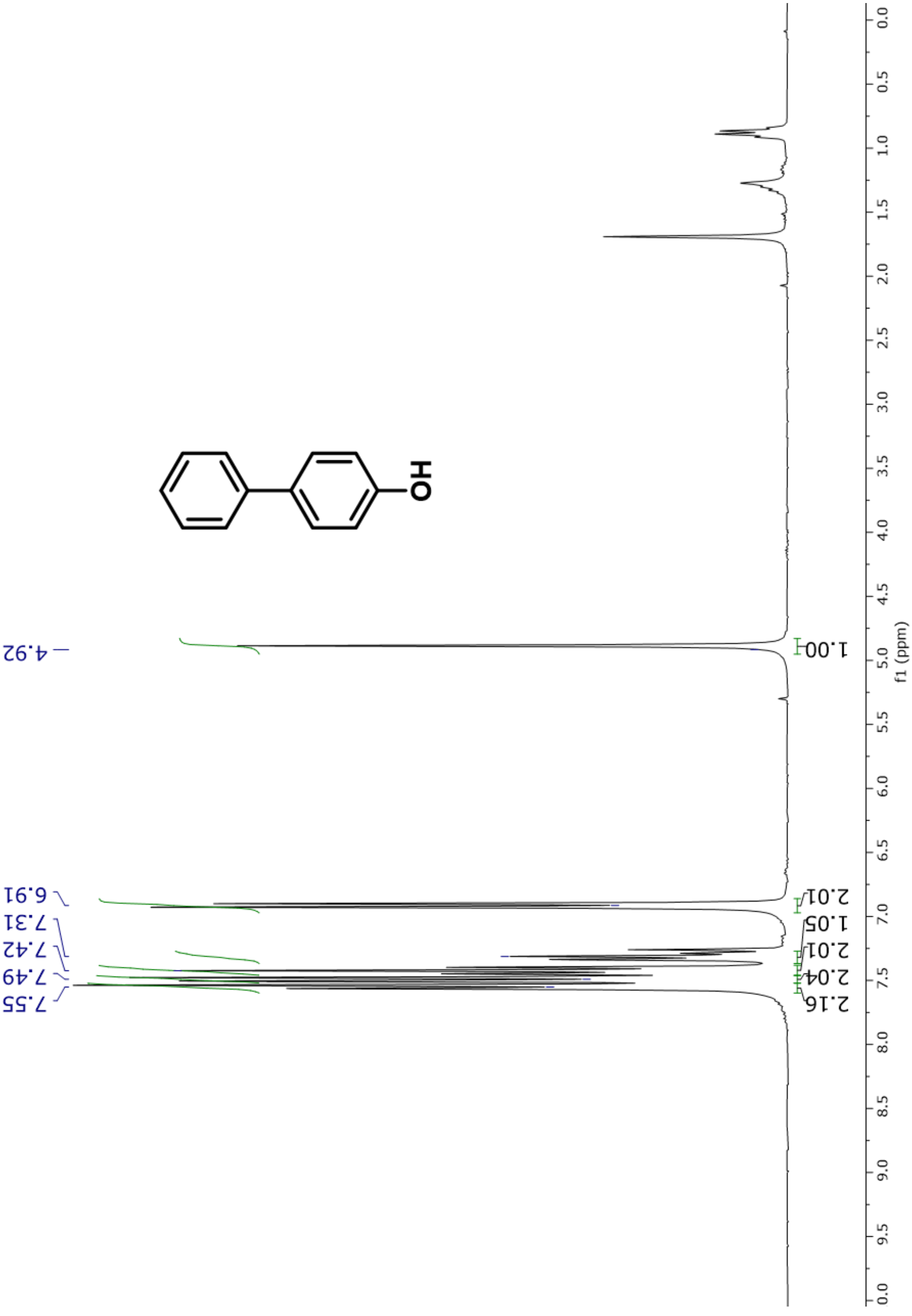
OH
4.92
6.91
7.31
7.42
7.49
7.55
1.00
2.01
1.05
2.01
2.04
2.16
f1 (ppm)

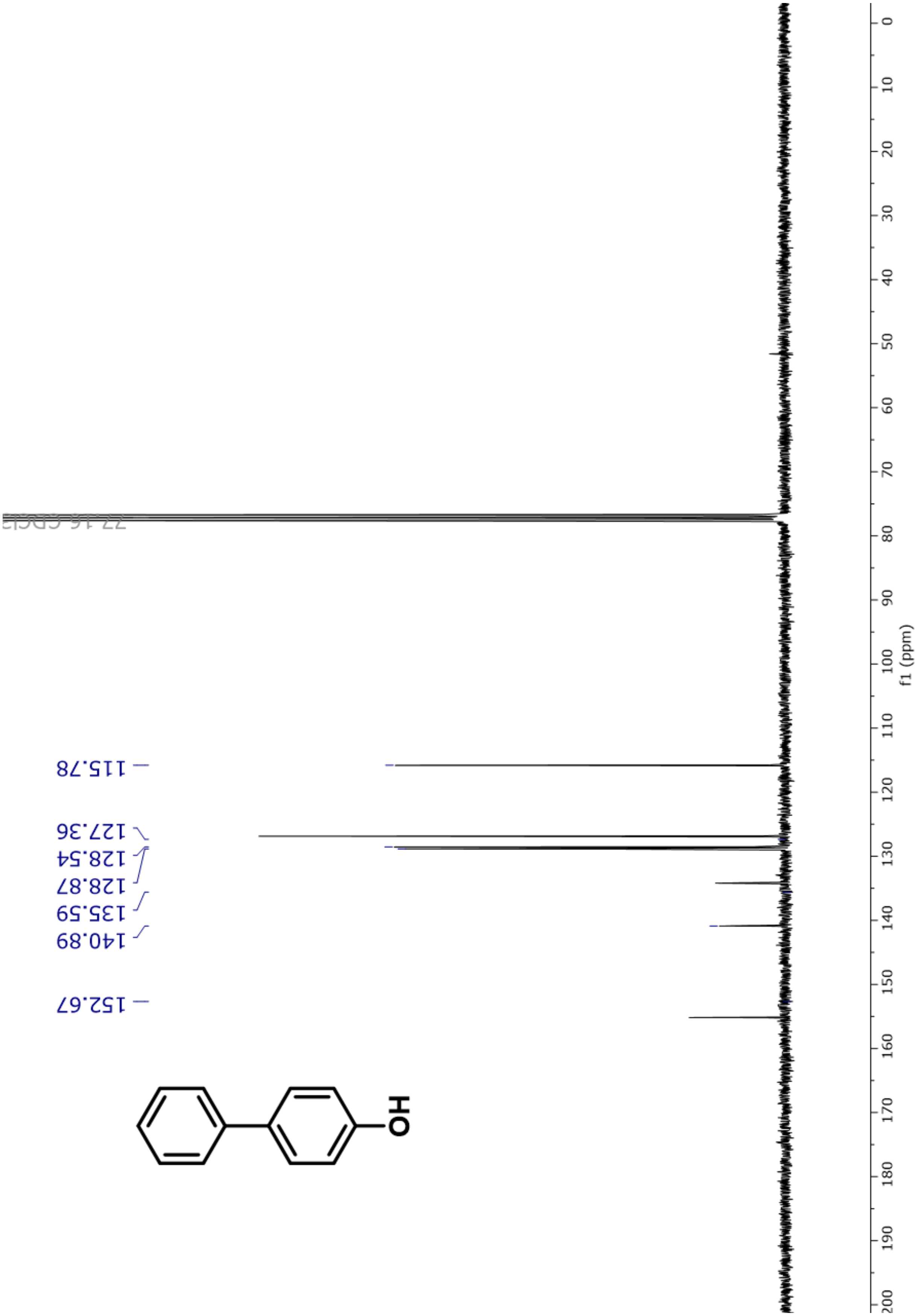
152.67
140.89
135.59
128.87
128.54
127.36
115.78
77.16 CDCl3
OH
f1 (ppm)
0
10
20
30
40
50
60
70
80
90
100
110
120
130
140
150
160
170
180
190
200

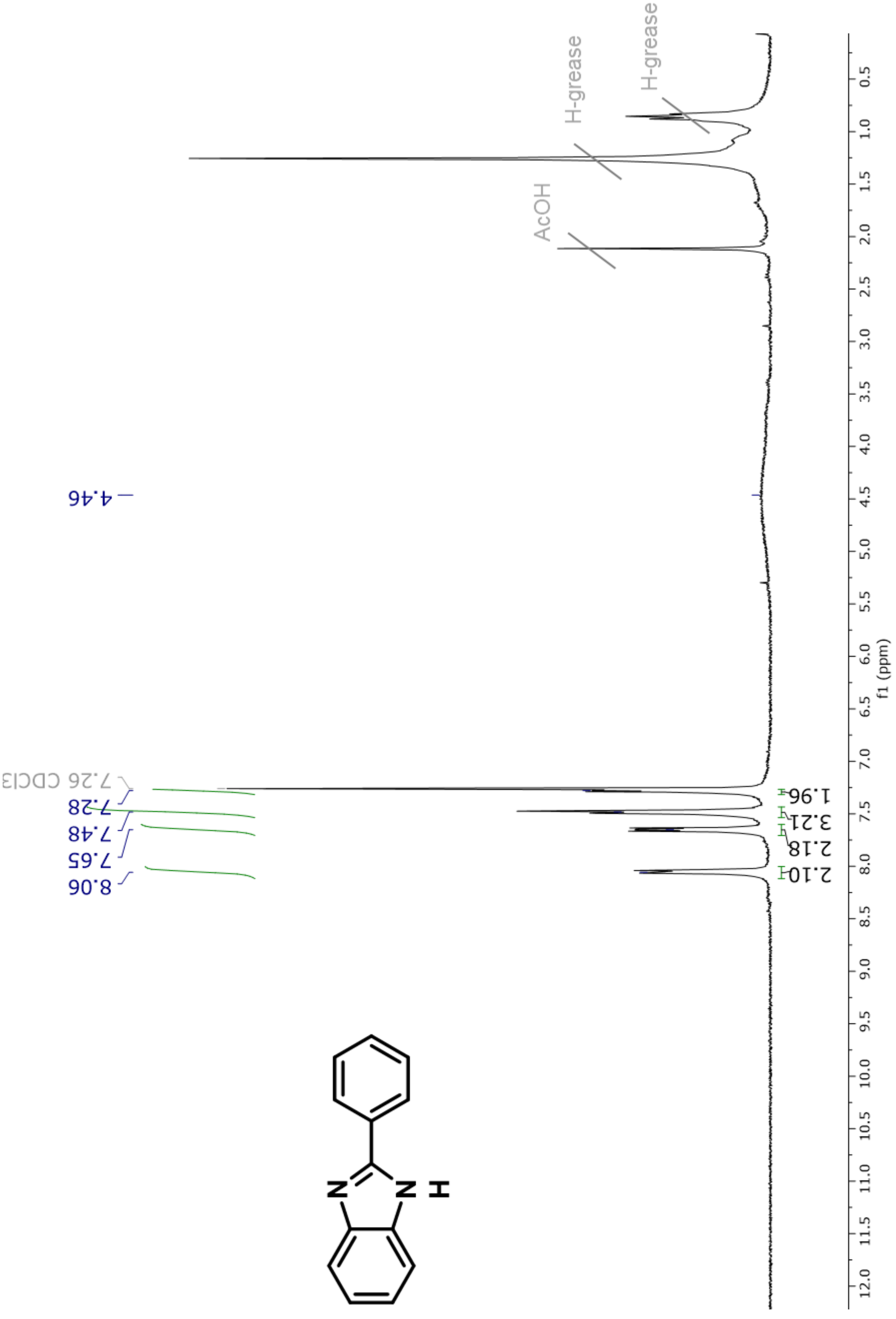
H-grease
H-grease
AcOH
4.46
7.26 CDCl3
7.28
7.48
7.65
8.06
1.96
3.21
2.18
2.10
f1 (ppm)
N
N
H

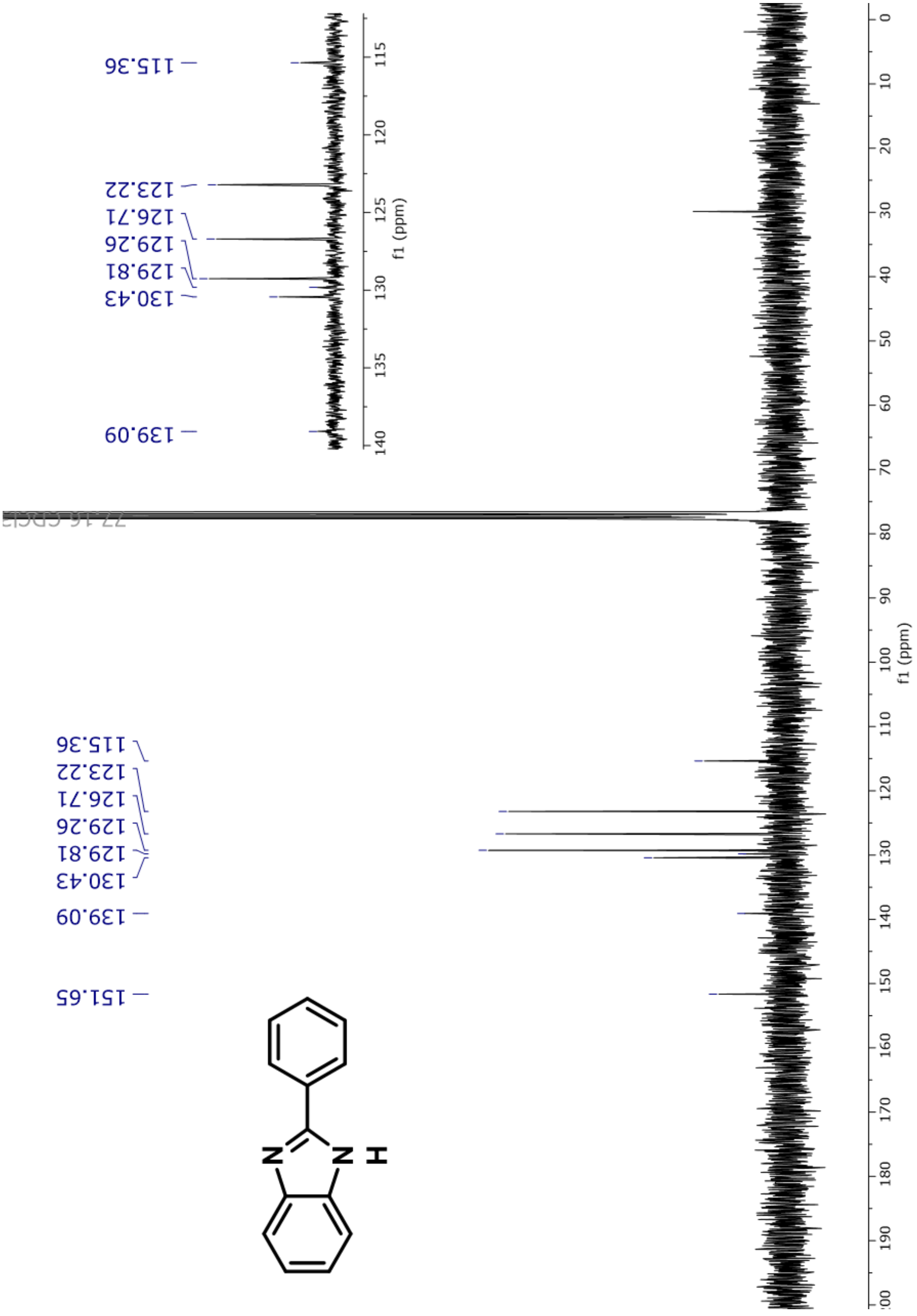
151.65
139.09
130.43
129.81
129.26
126.71
123.22
115.36
f1 (ppm)

Supporting Information References

[1] M. Frisch, G. Trucks, H. B. Schlegel, G. E. Scuseria, M. A. Robb, J. R. Cheeseman, G. Scalmani, V. Barone, B. Mennucci, G. Petersson, Gaussian, Inc., Wallingford CT, **2009**.

[2] A. D. Becke, *J. Chem. Phys.* **1993**, *98*, 5648-5652.

[3] a) T. Clark, J. Chandrasekhar, G. W. Spitznagel, P. V. Schleyer, *J. Comput. Chem.* **1983**, *4*, 294-301; b) A. D. McLean, G. S. Chandler, *J. Chem. Phys.* **1980**, *72*, 5639-5648.
